# Supplementary material for: Assessment of correlates of hand hygiene compliance among final year medical students: a cross-sectional study in the Netherlands
Source: BMJ Open. 2020 Feb 12;10(2):e029484. doi: 10.1136/bmjopen-2019-029484 (PMC7045092; doi:10.1136/bmjopen-2019-029484)
Supplement: Author's manuscript [file bmjopen-2019-029484.draft_revisions.pdf]

BMJ Open is committed to open peer review. As part of this commitment we make the peer review history of every article we publish publicly available.

When an article is published we post the peer reviewers' comments and the authors' responses online. We also post the versions of the paper that were used during peer review. These are the versions that the peer review comments apply to.

The versions of the paper that follow are the versions that were submitted during the peer review process. They are not the versions of record or the final published versions. They should not be cited or distributed as the published version of this manuscript.

BMJ Open is an open access journal and the full, final, typeset and author-corrected version of record of the manuscript is available on our site with no access controls, subscription charges or pay-per-view fees (http://bmjopen.bmj.com).

If you have any questions on BMJ Open's open peer review process please email info.bmjopen@bmj.com

BMJ Open

Assessment of correlates of hand hygiene compliance among final year medical students: a cross-sectional study

Journal:	*BMJ Open*
Manuscript ID	bmjopen-2019-029484
Article Type:	Research
Date Submitted by the Author:	28-Jan-2019
Complete List of Authors:	Erasmus, Vicki; Erasmus MC, Public Health<br>Otto, Suzie; Erasmus MC, Public Health<br>De Roos, E; Erasmus MC, Public Health<br>van Eijsden, Rianne; Erasmus MC, Public Health<br>Vos, Margreet; Erasmus MC Medical Center Rotterdam, Department of Medical Microbiology and Infectious diseases<br>Burdorf, Alex; Erasmus MC, Department of Public Health<br>VAN BEECK, Ed ; Erasmus MC, Public Health
Keywords:	hand hygiene, compliance, behavior, habit, medical students

SCHOLARONE™
Manuscripts

I, the Submitting Author has the right to grant and does grant on behalf of all authors of the Work (as defined in the below author licence), an exclusive licence and/or a non-exclusive licence for contributions from authors who are: i) UK Crown employees; ii) where BMJ has agreed a CC-BY licence shall apply, and/or iii) in accordance with the terms applicable for US Federal Government officers or employees acting as part of their official duties; on a worldwide, perpetual, irrevocable, royalty-free basis to BMJ Publishing Group Ltd ("BMJ") its licensees and where the relevant Journal is co-owned by BMJ to the co-owners of the Journal, to publish the Work in this journal and any other BMJ products and to exploit all rights, as set out in our licence.

The Submitting Author accepts and understands that any supply made under these terms is made by BMJ to the Submitting Author unless you are acting as an employee on behalf of your employer or a postgraduate student of an affiliated institution which is paying any applicable article publishing charge ("APC") for Open Access articles. Where the Submitting Author wishes to make the Work available on an Open Access basis (and intends to pay the relevant APC), the terms of reuse of such Open Access shall be governed by a Creative Commons licence – details of these licences and which Creative Commons licence will apply to this Work are set out in our licence referred to above.

Other than as permitted in any relevant BMJ Author's Self Archiving Policies, I confirm this Work has not been accepted for publication elsewhere, is not being considered for publication elsewhere and does not duplicate material already published. I confirm all authors consent to publication of this Work and authorise the granting of this licence.

For peer review only

Assessment of correlates of hand hygiene compliance among final year medical students: a cross-sectional study

Vicki Erasmus [1], Suzie J Otto [1], Emmely W. de Roos [1], Rianne van Eijsden [1], Margreet C Vos [2], Alex Burdorf [1], Ed F van Beeck [1]

[1] Department of Public Health, University Medical Center Rotterdam, 3000 CA Rotterdam, the Netherlands

[2] Department of Medical Microbiology and Infectious Diseases, University Medical Center Rotterdam, 3000 CA, Rotterdam, the Netherlands

Correspondence should be addressed to:

Vicki Erasmus

Erasmus University Medical Center

Department of Public Health

P.O. Box 2040

3000 CA Rotterdam

The Netherlands

Tel: +31 10 70 38777

v.erasmus@erasmusmc.nl

For peer review only

Key words: hand hygiene, compliance, behavior, habit, medical students

Word count: 2 605

Abstract

Objectives To identify the factors that influence hand hygiene compliance of final year medical students, using a theoretical behavioural framework.

Design Cross-sectional survey assessing self-reported compliance and its behavioural correlates.

Setting

Internships of medical students in the Netherlands.

Participants

322 Medical students of the Erasmus Medical Center were recruited over a period of 12 months during the internship Public health, which is the final compulsory internship after an 18 month rotation schedule in all major specialties.

Primary and secondary outcome measures

Behavioural factors influencing compliance to hand-hygiene were measured by means of a questionnaire based on the Theory of Planned Behaviour and Social Ecological Models . Multiple linear regression analysis was used to identify the effect of including attitudes, social norms, self-efficacy, knowledge, risk perception and habit on hand hygiene compliance.

Results

We included 313 students in the analysis (response of 97%). The behavioural model explained 40% of the variance in self-reported compliance (adjusted $R^2 = 0.40$). Hand hygiene compliance was strongly influenced by attitudes (perceived outcomes of preventive actions), self-efficacy (perception of the ability to perform hand hygiene at the clinical ward) and habit, but was not associated with knowledge and risk perception.

Conclusions

Targeting medical students' behaviour should focus on the empowerment of these juniors and provide them with evidence on the health benefits of prevention, rather than increasing their factual knowledge of procedures. Clinical teaching environments could help them form good patient safety habits during this vital phase of their career.

For peer review only - http://bmjopen.bmj.com/site/about/guidelines.xhtml

Article Summary

Strengths and limitations of this study

1. This study uses a hand hygiene questionnaire, based on insights from both the Theory of Planned Behaviour and Social Ecological Models.
2. The hand hygiene behaviour of medical students is investigated from a behavioural perspective in a large sample of students at the same stage of their internships.
3. The main limitations of this study are its cross-sectional design and the self-reported compliance with hand hygiene guidelines.

For peer review only

Introduction

Patient safety made its entrance into the fields of medical research and practice in the last decade of the twentieth century. [1-3] Ever since, patient safety has gained rising priority among health institutions, governments and insurance companies, who are all seeking to reduce the human and financial costs of preventable adverse events. [4] Prioritizing these events based on their impact and frequency of occurrence, shows that - next to surgical [5-7] and medication procedures [8 9] - infection prevention is a key element to improve patient safety.[10 11] In high income countries, 3.5% to 12% of all hospitalized patients contract one or more health care associated infections (HAI), while approximately 20-30% of patients in critical care are affected.[12] This high incidence of HAI not only accounts for prolonged hospital stay and preventable morbidity and mortality of patients and additional costs, but also enlarges the global threat to human health due to the emergence of multi-resistant bacteria by using antibiotics needed to treat these infections.[13 14]

Adequate hand hygiene behaviour by all medical professionals has been recognized as an eminent measure to reduce transmission of (multi-resistant) pathogens.[14] However, adherence to hand hygiene guideline, as with many quality improvement guidelines, remains low.[15] Medical professionals' patient safety practices, including their (non)adherence to hand hygiene guidelines, should be traced back to how future medical professionals are trained today.[16-19] The inclusion of medical students in patient safety initiatives is vital, because students shape their behavior and form habits during their internships, making them a priority group to be targeted in interventions.[20]

In the last decades, research into the behavioural factors influencing the hand hygiene behaviour of health care professionals has received growing attention.[21 22] Assessment of hand hygiene behavioral factors was often guided by behavioral theories, in particular the Theory of Planned Behavior (TPB),[23-27]. The TPB states that individual behaviour is influenced by attitudes, social norms (i.e. what people around you think and do) and self-efficacy (i.e. whether you feel that you are able to perform the behavior).[28] This theory has been used to explain the behavior of physicians and nurses, but not

For peer review only - http://bmjopen.bmj.com/site/about/guidelines.xhtml

the behavior of medical students yet. The few studies conducted so far into factors explaining hand hygiene compliance among medical students lacked a theoretical behavioral framework and were restricted to environmental factors such as access to facilities (low access associated with low compliance) and compliance of peers and superiors (low compliance of medical staff associated with low student compliance).[29 30] Therefore, to date knowledge is still limited on the behavioral factors that influence patient safety behaviors, including hand hygiene, of our next generation of physicians.[31] These insights can help clinical educators to promote patient safe behavior among medical students and thereby improve patient safety in the near future.

In this study we sought to identify correlates of the hand hygiene compliance of sixth-year medical students, using a theoretical behavioral framework.

Methods

Setting and participants

Over a period of 12 months we recruited a class of 322 medical interns. All students were enrolled in their last compulsory internship in Public Health at the Erasmus Medical Centre Rotterdam (a 1,320 bed university hospital). Every 2 weeks, a group of 10-15 students started their Public Health training and completed a questionnaire. Before this internship, the students followed an 18 month rotation schedule in all major specialties: internal medicine, surgery, pediatrics, gynaecology, neurology, psychiatry, ENT (Ear, Nose and Throat medicine), dermatology, general practice. The interns rotated among the university medical centre and 20 non-academic hospitals within the region South-West Netherlands. These institutions serve over 6.3 million people of various social and ethnic backgrounds in a mixed urban and rural area. In the second year of the study, undergraduate medical students follow the compulsory module ‘Basic Hygiene’ of the Unit Infection prevention (Erasmus MC), in which they are taught the principles of hand hygiene, among other.

For peer review only

All students were made aware that participation was voluntary and a verbal consent was requested and obtained from the participants. This study is part of the hand hygiene project, which was provided a waiver by institutional review board of Erasmus MC Rotterdam.

Behavioural theory and questionnaire

We developed the questionnaire used in this study for a larger national study on the determinants of hand hygiene compliance. This questionnaire is based on a translated version of the Hand hygiene Assessment Instrument (HAI)[32] with additional constructs identified by qualitative research among physicians, nurses and medical students.[16]

The HAI was translated into Dutch by two Dutch speakers and translated back into English by a native speaker to ensure the content had not changed.

The questionnaire is based on the TPB [28] and a number of additional constructs from the Social Ecological Model (SEM)[33] and the Habit Scale Index [34] were added. **Figure 1** shows our extended TPB model. *Behaviour* (in this case self-reported compliance, a behavior in previous internships, is influenced by *intention* (whether you intend or plan to comply with guidelines), which in turn is influenced by *attitudes* (beliefs of the importance of hand hygiene; outcome beliefs, i.e. expected outcomes of hand hygiene); *social norms* (referent beliefs, i.e. beliefs about how other people think about hand hygiene; descriptive norm, i.e. the perceived behaviour of others) and *self efficacy* (the perception of whether you think you could perform hand hygiene). We added the following additional constructs: *knowledge of the guidelines*, *risk perception* (chance of infection occurring; severity of infection for self (i.e. student) or patient), and *habit* (hand hygiene is something you do without thinking about it), measured with the Self-report index of Habit.[34] Since the internal consistency was good for each construct (Cronbachs $\alpha \geq .70$) we calculated average scores for use in further analysis (see **table 1**). All items, with the exception of the knowledge questions (measured by 5 true/false questions), were answered using 7 point Likert scales.

For peer review only - http://bmjopen.bmj.com/site/about/guidelines.xhtml

Self-reported compliance to hand hygiene guidelines, as outcome measure, was measured on a scale from 0 to 10 (never to always) for 13 potential hand hygiene situations (e.g. before touching a patient, before wound care, after patient contact).[32] For each respondent, we calculated an average score for use in further analysis.

Statistical analysis

We performed hierarchical multivariate linear regression analysis to identify the effects of the behavioural factors (independent variables) on self-reported compliance (dependent variable). The constructs were added to the model in 3 steps:

1) knowledge and risk perception,

2) all factors of step 1, with the addition of attitude, social norms and self-efficacy, and

3) all factors of step 2, with the addition of habit.

Comparison of self-reported compliance scores between male and female students was done by means of t-test for between group comparisons.

Results

Demographic data

In total, 313 (97%) students filled in at least 75% of the questionnaire and were included in the analysis. The students had an average age of 25.3 years (Standard Deviation (SD) 2.9), and 201 (64%) of the students was female, which is representative for the male-female ratio of medical students in the Netherlands.[35]

Self-reported compliance

The average self-reported compliance was 8.1 on a 10 point scale (SD .96). This measure was 8.2 (SD .92) for females and 7.8 (SD .99) for males. This difference was statistically significant ($P<.05$). Self-

For peer review only - http://bmjopen.bmj.com/site/about/guidelines.xhtml

reported compliance ranged from 4.3 (when resuming care after an interruption to 9.8 (after direct contact with body fluids).

Behavioural correlates

Table 2 shows the behavioural correlates associated with hand hygiene compliance in medical students. The regression coefficient β indicates the slope of the regression-line, and gives the average increase of compliance when the variable increases by 1.

Knowledge of guidelines and risk perception explained 4.3% of the variance of self-reported compliance (adjusted R^2 = .043) (model 1**)**.. However, the contribution of these factors to self-reported hand hygiene compliance was not statistically significant.

The addition of attitude, social norms and self-efficacy (model 2), resulted in an explained variance of 27%: (R^2 =.270), with perceived outcomes (an element of attitude) (β=.231, *P*<.001) and self-efficacy (β=.306, *P*<.001) showing a statistically significant association with self-reported compliance.

In model 3, the addition of habit resulted in an explained variance of 40% (adjusted R^2=.401), with habit showing a strong and statistically significant association with self-reported compliance, (β=.471, *P*<.001). The associations of perceived outcomes and self-efficacy were somewhat weakened in this final model , but both remained statistically significant at the 5% level.

Discussion

The results of this study show that hand hygiene behaviour of final year medical students, i.e. the new generation of physicians, is most strongly influenced by habit, perceived outcomes of hand hygiene and whether students feel they have the ability to perform hand hygiene in practice. Our extended behavioural model, which included attitudes, social norms, self-efficacy, knowledge, risk perception and habit, was able to explain a substantial part of the variance in self-reported compliance (adjusted R^2=.401).

For peer review only - http://bmjopen.bmj.com/site/about/guidelines.xhtml

One strength of our study is that we were able to include a full class of all medical students during one year, with a response of 97%. Students were approached to fill out the questionnaire after they had completed an 18 month rotation schedule of 9 specialties in both teaching and non-teaching facilities in a mixed urban/rural area. This class therefore had recently experienced a large number of hospital settings and patient types. After graduation, the students may select any clinical or non-clinical specialty, and our study population therefore represents juniors that will continue their career within a broad spectrum of medical disciplines. A second strength of this study is that we used a hand hygiene questionnaire, based on combined insights from the Theory of Planned Behaviour and Social Ecological Models.

But the use of self-reported compliance as the primary outcome measure and lack of observational data form one of this study's limitations, and we are therefore only able to base our model on self-reported behaviour. In the setting of an internship in public health, where this study was conducted, opportunities for hand hygiene are almost absent and directly observing hand hygiene compliance in the multitude of very diverse medical institutions during the preceding rotation schedule would not have yielded comparable data. This rotation schedule also resulted in the use of a cross-sectional design, which restricts conclusions on causality. A longitudinal study would resolve this restriction but would be arduous due to the rotations and most likely result in a high loss due to follow up and a much lower response rate as a result. Therefore the use of self-reported data in a cross-sectional design was the best option in our case.

A second limitation of our study arises from our inability to explore the influence of cultural factors in our analyses. International patient safety experts have addressed the need to tackle not only individual change but also organizational change in order to improve patient safety culture.[1] A poor safety culture has been found to be associated with adverse events and a substantial improvement requires a culture of safety within the organization.[36] We would therefore have liked to include the

For peer review only - http://bmjopen.bmj.com/site/about/guidelines.xhtml

construct culture in our model, but due to the large number of wards within different hospitals that students worked on (and therefore large number of cultures they experienced) we were unable to investigate its effects in this study. Culture could prove a valuable addition to the model and explain an additional part of the behaviour of medical students. The influence of culture should be further investigated in a different study design focusing on the observed compliance of interns of specific units, hospitals and/or specialties.

As a third limitation it must be mentioned that, although we used an extended version of the Theory of Planned Behavior, potential correlates of hand hygiene compliance could be omitted. Recent studies have used more comprehensive models, such as the Theory Domains Framework [37] and the Health Action Process Approach [38] to explain hand hygiene compliance of physicians and nurses and their application in medical students could be considered. Although the addition of the Habit Scale Index did add an extra 13% explained variance, indicating hand hygiene is a strongly habitual behavior, and interventions to improve it should not only focus on volitional construct

Only a few studies on the observed hand hygiene compliance of medical students have been conducted so far, mostly looking at other factors than student behavior (e.g. facilities), limiting a straightforward comparison of the results presented here [29 30]. We found that in addition to external factors such as access to facilities and compliance of superiors, student- related behavioural factors make their contribution as well.

Previously positive attitudes towards hand hygiene, and in particular positive beliefs about the outcome of performing hand hygiene have been found to be significantly associated with hand hygiene compliance of nurses and physicians.[22 34 39], similar to the results we found here for medical students. The influence of habit [34] on hand hygiene behaviour had not previously been reported, and we found a strong association between habit and the self-reported hand hygiene behaviour of

For peer review only - http://bmjopen.bmj.com/site/about/guidelines.xhtml

medical students. A study from the UK found that the observed hand hygiene guideline compliance of medical students in an examinational setting was extremely low, even in the presence of 'Wash Your Hands' signs.[40] A hand hygiene intervention after the SARS (Severe acute respiratory syndrome) outbreak in Asia had good results, and was found to be related to a higher level of perceived risk; risk perception was not found to be significantly associated with hand hygiene compliance in this study, although this difference could be a result of the extreme situational circumstances during the SARS outbreak.[41]

Much of the behaviour of medical students is based on the behaviour of the role models (often residents) they encounter during their clinical phase, and not on what they have learned during their preclinical phase.[17 42 43] Once they reach their internship-phase, medical students are confronted with and adapt themselves to a culture of non-adherence. This effect is also present among residents, as with a senior member of the team performing hand hygiene, the hand hygiene compliance of residents increases significantly, but overall compliance of residents is as low as their qualified colleagues (< 40%).[42] It is therefore essential to break the vicious circle and one way to do this is by preparing medical students for the incongruencies they will encounter in clinical practice and increase their coping skills.

It is increasingly recognized that patient safety should be improved through education.[18 31] Medical students indicate themselves that more education on patient safety and especially hand hygiene is necessary.[44] Residents state that medical mistakes could be prevented with more education on the matter.[45] Our results show that traditional educational methods focusing on knowledge improvement are not the way to go in order to stimulate better patient safety behaviours, such as hand hygiene compliance. Similar as for physicians and nurses it is essential to target medical students with interventions tailored to the major modifiable determinants of non-compliance. Targeting medical students' behaviour should focus on the empowerment of these juniors, rather

For peer review only - http://bmjopen.bmj.com/site/about/guidelines.xhtml

than increasing their factual knowledge of procedures. Insights from the behavioural sciences may be useful to increase the self-efficacy of this important target group. Interventions using the concept of action planning have been successful in several settings [46], including hospital care [47] and seem to be promising in this context.

Adequate hand hygiene can lead to a reduced rate of health care associated infections and a drop in adverse events, morbidity and mortality. Application of behavioural insights can lead to patient safety improvements throughout health care, and ultimately to safer hospitals. Breaking through the culture of non-adherence is the first step to achieving this goal.

In conclusion, targeting medical students' behaviour should focus on the empowerment of these juniors and provide them with evidence on the health benefits of prevention, rather than increasing their factual knowledge of procedures. Clinical teaching environments could help them form good patient safety habits during this vital phase of their career.

For peer review only

For peer review only - http://bmjopen.bmj.com/site/about/guidelines.xhtml

Acknowledgments

The authors wish to thank medical students for their participation in this study.

Authors contribution

The study was conceived and designed by VE and EB. Data collection was performed by VE and AE and analyzed by VE. All authors were involved in the interpretation of the data. VE wrote the first draft of the manuscript. All authors critically revised the manuscript, and read and approved the final manuscript.

For peer review only

Funding

This work was supported by the Netherlands Organisation for Health Research and Development (*ZonMW*); grant number 50-50115-96-632.

Competing interests

The authors declare that they have no competing interests.

Patient consent

Not required.

Data sharing

No additional data are available.

For peer review only - http://bmjopen.bmj.com/site/about/guidelines.xhtml

References

1. Kohn LT, Corrigan JM, Donaldson MS, editors. *To err is human: Building a safer health system*. Washington D.C: National Academy Press, 2000.
2. Reason J. Human error: models and management. Brit Med J 2000;**320**(7237):768-70.
3. Helmreich RL. On error management: lessons from aviation. Brit Med J 2000;**320**(7237):781-85.
4. Wise J. MPs attack NHS for putting finances and targets ahead of patient safety. Brit Med J 2009;**339**.
5. Gawande AA, Thomas EJ, Zinner MJ, et al. The incidence and nature of surgical adverse events in Colorado and Utah in 1992. Surgery 1999;**126**(1):66-75.
6. Weiser TG, Makary MA, Haynes AB, et al. Standardised metrics for global surgical surveillance. Lancet 2009;**374**(9695):1113-7.
7. Zegers M, de Bruijne MC, de Keizer B, et al. The incidence, root-causes, and outcomes of adverse events in surgical units: implication for potential prevention strategies. Patient Saf Surg 2011;**5**:13.
8. Bates DW, Cullen DJ, Laird N, et al. Incidence of adverse drug events and potential adverse drug events. Implications for prevention. ADE Prevention Study Group. JAMA 1995;**274**(1):29-34.
9. Dequito AB, Mol PG, van Doormaal JE, et al. Preventable and non-preventable adverse drug events in hospitalized patients: a prospective chart review in the Netherlands. Drug Safety 2011;**34**(11):1089-100.
10. Burke JP. Infection control - a problem for patient safety. N Engl J Med 2003;**348**(7):651-6.
11. Pittet D, Donaldson L. Clean care is safer care: the first global challenge of the WHO World Alliance for Patient Safety. Am J Infect Control 2005;**33**(8):476-9.
12. World Health Organization. Chapter 3. The burden of endemic health care-associated infection in high-income countries. In: Report on the burden of endemic health care-associated infection worldwide. Geneva: WHO Press, 2011:12-15.
13. Donaldson L. Dirty hands: the human cost. CMO Annual Report 2006 2006.
14. World Health Organization. World Alliance for Patient Safety. The Global Patient Safety Challenge 2005–2006. Clean Care is Safer Care. Geneva: WHO Press, 2005.
15. Erasmus V, Daha TJ, Brug H, et al. Systematic review of studies on compliance with hand hygiene guidelines in hospital care. Infect Control Hosp Epidemiol 2010;**31**(3):283-94.
16. Erasmus V, Brouwer W, van Beeck EF, et al. A qualitative exploration of reasons for poor hand hygiene among hospital workers: lack of positive role models and of convincing evidence that hand hygiene prevents cross-infection. Infect Control Hosp Epidemiol 2009;**30**(5):415-9.
17. Lankford MG, Zembower TR, Trick WE, et al. Influence of role models and hospital design on hand hygiene of healthcare workers. Emerg Infect Dis 2003;**9**(2):217-23.
18. Paterson-Brown S. Improving patient safety through education. BMJ 2011;**342**:d214.
19. Snow M, White GL, Jr., Alder SC, et al. Mentor's hand hygiene practices influence student's hand hygiene rates. Am J Infect Control 2006;**34**(1):18-24.
20. Wakefield JG, McLaws ML, Whitby M, et al. Patient safety culture: factors that influence clinician involvement in patient safety behaviours. Qual Saf Health Care 2010;**19**(6):585-91.

For peer review only - http://bmjopen.bmj.com/site/about/guidelines.xhtml

21. Pittet D. Improving compliance with hand hygiene in hospitals. Infect Control Hosp Epidemiol 2000;**21**(6):381-6.
22. Whitby M, Pessoa-Silva CL, McLaws ML, et al. Behavioural considerations for hand hygiene practices: the basic building blocks. J Hosp Infect 2007;**65**(1):1-8.
23. O'Boyle CA, Henly SJ, Larson E. Understanding adherence to hand hygiene recommendations: the theory of planned behavior. Am J Infect Control 2001;**29**(6):352-60.
24. Jenner EA, Watson PW, Miller L, et al. Explaining hand hygiene practice: an extended application of the Theory of Planned Behaviour. Psychol Health Med 2002;**7**:311-26.
25. Sax H, Uckay I, Richet H, et al. Determinants of good adherence to hand hygiene among healthcare workers who have extensive exposure to hand hygiene campaigns. Infect Control Hosp Epidemiol 2007;**28**(11):1267-74.
26. De Wandel D, Maes L, Labeau S, et al. Behavioral determinants of hand hygiene compliance in intensive care units. Am J Crit Care 2010;**19**(3):230-9.
27. White KM, Jimmieson NL, Obst PL, et al. Using a theory of planned behaviour framework to explore hand hygiene beliefs at the '5 critical moments' among Australian hospital-based nurses. BMC Health Serv Res 2015;**15**:59.
28. Ajzen J. The theory of planned behavior. Organizational behavior and human decision processes 1991;**50**:179-211.
29. Hunt DC, Mohammudally A, Stone SP, et al. Hand-hygiene behaviour, attitudes and beliefs in first year clinical medical students. J Hosp Infect 2005;**59**(4):371-3.
30. Polacco MA, Shinkunas L, Perencevich EN, et al. See one, do one, teach one: Hand hygiene attitudes among medical students, interns, and faculty. American Journal of Infection Control 2015;**43**(2):159-61.
31. Humphreys H, Richards J. Undergraduate and postgraduate medical education on the prevention and control of healthcare-associated infection. More progress is needed. International Journal of Infection Control 2011:5.
32. O'Boyle C A, Henly SJ, Duckett LJ. Nurses' motivation to wash their hands: a standardized measurement approach. Appl Nurs Res 2001;**14**(3):136-45.
33. Bronfenbrenner U. *The ecology of human development*. Cambridge, MA: Harvard University Press, 1979.
34. Verplanken B, Aarts H. Habit, Attitude and Planned Behaviour: Is Habit an Empty Construct or an Interesting Case of Goal-Directed Automaticity? . European Review of Social Psychology 1999;**10**:101-34.
35. van der Velden LF, Hingstman L, Heiligers PJ, et al. [Increasing number of women in medicine: past, present and future] Toenemend percentage vrouwen in de geneeskunde: verleden, heden en toekomst. Ned Tijdschr Geneeskd 2008;**152**(40):2165-71.
36. Nieva VF, Sorra J. Safety culture assessment: a tool for improving patient safety in healthcare organizations. Qual Saf Health Care 2003;**12 Suppl 2**:ii17-23.
37. Fuller C, Besser S, Savage J, et al. Application of a theoretical framework for behavior change to hospital workers' real-time explanations for noncompliance with hand hygiene guidelines. Am J Infect Control 2014;**42**(2):106-10.
38. von Lengerke T, Lutze B, Graf K, et al. Psychosocial determinants of self-reported hand hygiene behaviour: a survey comparing physicians and nurses in intensive care units. J Hosp Infect 2015;**91**(1):59-67.

For peer review only - http://bmjopen.bmj.com/site/about/guidelines.xhtml

39. Pittet D, Simon A, Hugonnet S, et al. Hand hygiene among physicians: performance, beliefs, and perceptions. Ann Intern Med 2004;**141**(1):1-8.
40. Feather A, Stone SP, Wessier A, et al. 'Now please wash your hands': the handwashing behaviour of final MBBS candidates. J Hosp Infect 2000;**45**(1):62-4.
41. Wong TW, Tam WW. Handwashing practice and the use of personal protective equipment among medical students after the SARS epidemic in Hong Kong. Am J Infect Control 2005;**33**(10):580-6.
42. Rome M, Sabel A, Price CS, et al. Hand hygiene compliance. J Hosp Infect 2007;**65**(2):173.
43. Walton MM. Hierarchies: the Berlin Wall of patient safety. Qual Saf Health Care 2006;**15**(4):229-30.
44. Mann CM, Wood A. How much do medical students know about infection control? J Hosp Infect 2006;**64**(4):366-70.
45. Varkey P, Karlapudi S, Rose S, et al. A patient safety curriculum for graduate medical education: results from a needs assessment of educators and patient safety experts. Am J Med Qual 2009;**24**(3):214-21.
46. Gollwitzer PM, Sheeran P. Implementation intentions and goal achievement: a meta-analysis of effects and processes. Advances in Experimental Social Psychology 2006;**38**:69-119.
47. Erasmus V, Kuperus MN, Richardus JH, et al. Improving hand hygiene behaviour of nurses using action planning: a pilot study in the intensive care unit and surgical ward. J Hosp Infect 2010;**76**(2):161-4.

For peer review only

For peer review only - http://bmjopen.bmj.com/site/about/guidelines.xhtml

Table 1: Constructs of the questionnaire on behavioural determinants of hand hygiene with example questions and internal consistency (Cronbach's α)

Construct	# items	Mean (SD)	Cronbach's α	Example
**Knowledge**	5	4.3 (.78)	-	5 true/false questions about factual knowledge
**Risk perception**:				
Chance	3	5.6 (1,7)	0.76	How big is the chance that an infection will occur
Severity self	1	5.6 (2.3)	-	How severe will the consequences of an infection be for myself
Severity patient	1	7.2 (1.5)	-	How severe will the consequences of an infection be for my patient
**Attitudes**:				
Beliefs about hand hygiene	8	5.3 (.81)	0.76	Hand hygiene is something I find important
Perceived outcomes	5	5.0 (.98)	0.78	If I follow that hand hygiene guidelines my patients will develop fewer infections
**Social norms**:				
Referent beliefs	4	4.7 (1.2)	0.91	My superior thinks that I should always follow the hand hygiene guidelines
Descriptive norm	8	3.5 (.81)	0.73	My colleagues always follow the hand hygiene guidelines
**Self-efficacy**	11	4.8 (.94)	0.89	I am certain that I will be able to follow the hand hygiene guidelines
**Habit**	12	4.7 (1.1)	0.95	Following the hand hygiene guidelines is something I do without thinking about it

For peer review only

Table 2: Behavioural correlates of hand hygiene compliance of medical students (n=313)

	Model 1		Model 2			Model 3		
	β	R^2	B	R^2	$R^{2change}$	β	R^2	$R^{2change}$
		.043		.270	.189		.401	.131
Knowledge	.081		.044			.063		
Risk perception:								
Chance	.101		.049			.010		
Severity self	.105		.041			.002		
Severity patient	.102		.036			.019		
Attitude:								
Beliefs			.103			-.026		
Perceived outcomes			.231***			.174**		
Social norms:								
Referent beliefs			.003			-.001		
Descriptive norm			.063			.037		
Self efficacy			.306***			.138*		
Habit						.471***		

* P<.05; ** P<.01; *** P < .001

Model 1: knowledge + risk perception

Model 2: knowledge + risk perception + attitudes + social norms + self efficacy

Model 3: knowledge + risk perception + attitudes + social norms + self efficacy + habit

For peer review only

For peer review only - http://bmjopen.bmj.com/site/about/guidelines.xhtml

Figure

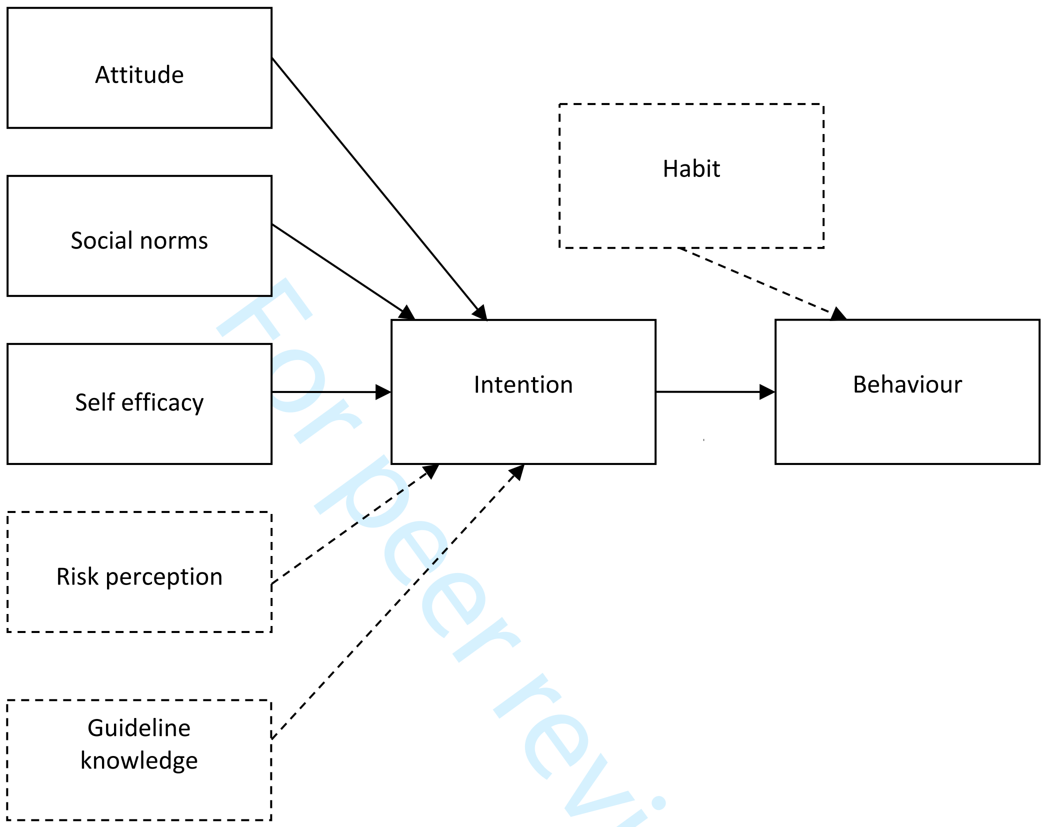

Figure 1: Extended Theory of Planned Behaviour model. Constructs in dashed boxes have been added.

For peer review only

Reporting checklist for cross sectional study.

Based on the STROBE cross sectional guidelines.

Instructions to authors

Complete this checklist by entering the page numbers from your manuscript where readers will find each of the items listed below.

Your article may not currently address all the items on the checklist. Please modify your text to include the missing information. If you are certain that an item does not apply, please write "n/a" and provide a short explanation.

Upload your completed checklist as an extra file when you submit to a journal.

In your methods section, say that you used the STROBE cross sectional reporting guidelines, and cite them as:

von Elm E, Altman DG, Egger M, Pocock SJ, Gotzsche PC, Vandenbroucke JP. The Strengthening the Reporting of Observational Studies in Epidemiology (STROBE) Statement: guidelines for reporting observational studies.

		Reporting Item	Page Number
Title	#1a	Indicate the study's design with a commonly used term in the title or the abstract	1
Abstract	#1b	Provide in the abstract an informative and balanced summary of what was done and what was found	2

For peer review only

Background / rationale	#2	Explain the scientific background and rationale for the investigation being reported	4
Objectives	#3	State specific objectives, including any prespecified hypotheses	5
Study design	#4	Present key elements of study design early in the paper	5
Setting	#5	Describe the setting, locations, and relevant dates, including periods of recruitment, exposure, follow-up, and data collection	5
Eligibility criteria	#6a	Give the eligibility criteria, and the sources and methods of selection of participants.	5
	#7	Clearly define all outcomes, exposures, predictors, potential confounders, and effect modifiers. Give diagnostic criteria, if applicable	6
Data sources / measurement	#8	For each variable of interest give sources of data and details of methods of assessment (measurement). Describe comparability of assessment methods if there is more than one group. Give information separately for for exposed and unexposed groups if applicable.	5-6
Bias	#9	Describe any efforts to address potential sources of bias	n/a
Study size	#10	Explain how the study size was arrived at	5
Quantitative variables	#11	Explain how quantitative variables were handled in the analyses. If applicable, describe which groupings were chosen, and why	7

For peer review only

Statistical methods	#12a	Describe all statistical methods, including those used to control for confounding	7
	#12b	Describe any methods used to examine subgroups and interactions	n/a
	#12c	Explain how missing data were addressed	n/a
	#12d	If applicable, describe analytical methods taking account of sampling strategy	n/a
	#12e	Describe any sensitivity analyses	n/a
Participants	#13a	Report numbers of individuals at each stage of study—eg numbers potentially eligible, examined for eligibility, confirmed eligible, included in the study, completing follow-up, and analysed. Give information separately for for exposed and unexposed groups if applicable.	7
	#13b	Give reasons for non-participation at each stage	n/a
	#13c	Consider use of a flow diagram	n/a
Descriptive data	#14a	Give characteristics of study participants (eg demographic, clinical, social) and information on exposures and potential confounders. Give information separately for exposed and unexposed groups if applicable.	7
	#14b	Indicate number of participants with missing data for each variable of interest	n/a
Outcome data	#15	Report numbers of outcome events or summary measures.	7-8, 19

For peer review only

		Give information separately for exposed and unexposed groups if applicable.	
Main results	#16a	Give unadjusted estimates and, if applicable, confounder-adjusted estimates and their precision (eg, 95% confidence interval). Make clear which confounders were adjusted for and why they were included	n/a
	#16b	Report category boundaries when continuous variables were categorized	n/a
	#16c	If relevant, consider translating estimates of relative risk into absolute risk for a meaningful time period	n/a
Other analyses	#17	Report other analyses done—e.g., analyses of subgroups and interactions, and sensitivity analyses	n/a
Key results	#18	Summarise key results with reference to study objectives	8
Limitations	#19	Discuss limitations of the study, taking into account sources of potential bias or imprecision. Discuss both direction and magnitude of any potential bias.	9-10
Interpretation	#20	Give a cautious overall interpretation considering objectives, limitations, multiplicity of analyses, results from similar studies, and other relevant evidence.	12
Generalisability	#21	Discuss the generalisability (external validity) of the study results	10-11
Funding	#22	Give the source of funding and the role of the funders for the present study and, if applicable, for the original study on which	13

For peer review only

the present article is based

The STROBE checklist is distributed under the terms of the Creative Commons Attribution License CC-BY. This checklist was completed on 12. December 2018 using http://www.goodreports.org/, a tool made by the EQUATOR Network in collaboration with Penelope.ai

For peer review only

BMJ Open

Assessment of correlates of hand hygiene compliance among final year medical students: a cross-sectional study

Journal:	*BMJ Open*
Manuscript ID	bmjopen-2019-029484.R1
Article Type:	Original research
Date Submitted by the Author:	04-Nov-2019
Complete List of Authors:	Erasmus, Vicki; Erasmus MC, Public Health<br>Otto, Suzie; Erasmus MC, Public Health<br>De Roos, E; Erasmus MC, Public Health<br>van Eijsden, Rianne; Erasmus MC, Public Health<br>Vos, Margreet; Erasmus MC Medical Center Rotterdam, Department of Medical Microbiology and Infectious diseases<br>Burdorf, Alex; Erasmus MC, Department of Public Health<br>VAN BEECK, Ed ; Erasmus MC, Public Health
**Primary Subject Heading**:	Medical education and training
Secondary Subject Heading:	Public health
Keywords:	hand hygiene, compliance, behavior, habit, medical students

SCHOLARONE™
Manuscripts

I, the Submitting Author has the right to grant and does grant on behalf of all authors of the Work (as defined in the below author licence), an exclusive licence and/or a non-exclusive licence for contributions from authors who are: i) UK Crown employees; ii) where BMJ has agreed a CC-BY licence shall apply, and/or iii) in accordance with the terms applicable for US Federal Government officers or employees acting as part of their official duties; on a worldwide, perpetual, irrevocable, royalty-free basis to BMJ Publishing Group Ltd ("BMJ") its licensees and where the relevant Journal is co-owned by BMJ to the co-owners of the Journal, to publish the Work in this journal and any other BMJ products and to exploit all rights, as set out in our licence.

The Submitting Author accepts and understands that any supply made under these terms is made by BMJ to the Submitting Author unless you are acting as an employee on behalf of your employer or a postgraduate student of an affiliated institution which is paying any applicable article publishing charge ("APC") for Open Access articles. Where the Submitting Author wishes to make the Work available on an Open Access basis (and intends to pay the relevant APC), the terms of reuse of such Open Access shall be governed by a Creative Commons licence – details of these licences and which Creative Commons licence will apply to this Work are set out in our licence referred to above.

Other than as permitted in any relevant BMJ Author's Self Archiving Policies, I confirm this Work has not been accepted for publication elsewhere, is not being considered for publication elsewhere and does not duplicate material already published. I confirm all authors consent to publication of this Work and authorise the granting of this licence.

For peer review only

Assessment of correlates of hand hygiene compliance among final year medical students: a cross-sectional study

Vicki Erasmus [1], Suzie J Otto [1], Emmely W. de Roos [1], Rianne van Eijsden [1], Margreet C Vos [2], Alex Burdorf [1], Ed F van Beeck [1]

[1] Department of Public Health, University Medical Center Rotterdam, 3000 CA Rotterdam, the Netherlands

[2] Department of Medical Microbiology and Infectious Diseases, University Medical Center Rotterdam, 3000 CA, Rotterdam, the Netherlands

Correspondence should be addressed to:

Vicki Erasmus

Erasmus University Medical Center

Department of Public Health

P.O. Box 2040

3000 CA Rotterdam

The Netherlands

Tel: +31 10 70 38777

v.erasmus@erasmusmc.nl

Key words: hand hygiene, compliance, behavior, habit, medical students

Word count: 3 038

For peer review only

Abstract

Objectives To identify the factors that influence the hand hygiene compliance of final year medical students, using a theoretical behavioural framework.

Design Cross-sectional survey assessing self-reported compliance and its behavioural correlates.

Setting

Internships of medical students in the Netherlands.

Participants

322 Medical students of the Erasmus Medical Center were recruited over a period of 12 months during the Public Health internship, which is the final compulsory internship after an 18 month rotation schedule in all major specialties.

Primary and secondary outcome measures

Behavioural factors influencing compliance to hand hygiene guidelines were measured by means of a questionnaire based on the Theory of Planned Behaviour and Social Ecological Models. Multiple linear regression analysis was used to identify the effect of including attitudes, social norms, self-efficacy, knowledge, risk perception and habit on hand hygiene compliance.

Results

We included 313 students in the analysis (response rate 97%). The behavioural model explained 40% of the variance in self-reported compliance (adjusted R2 = 0.40). Hand hygiene compliance was strongly influenced by attitudes (perceived outcomes of preventive actions), self-efficacy (perception of the ability to perform hand hygiene at the clinical ward) and habit, but was not associated with knowledge and risk perception.

Conclusions

Targeting medical students' behaviour should focus on the empowerment of these juniors and provide them with evidence on the health benefits of prevention, rather than increasing their factual knowledge of procedures. Clinical teaching environments could help them form good patient safety habits during this vital phase of their career.

For peer review only - http://bmjopen.bmj.com/site/about/guidelines.xhtml

Article Summary

Strengths and limitations of this study

1. This study uses a hand hygiene questionnaire, based on insights from both the Theory of Planned Behaviour and Social Ecological Models.
2. The hand hygiene behaviour of medical students is investigated from a behavioural perspective in a large sample of students at the same stage of their internships.
3. The main limitations of this study are its cross-sectional design and the self-reported compliance with hand hygiene guidelines.

For peer review only

For peer review only - http://bmjopen.bmj.com/site/about/guidelines.xhtml

Introduction

Patient safety made its entrance into the fields of medical research and practice in the last decade of the twentieth century.[1-3] Ever since, patient safety has become a rising priority among health institutions, governments and insurance companies, who are all seeking to reduce the human and financial costs of preventable adverse events.[4] Prioritizing these events based on their impact and frequency of occurrence, shows that - next to surgical [5-7] and medication procedures [8 9] - infection prevention is a key element to improve patient safety.[10 11] In high income countries, 3.5% to 12% of all hospitalized patients contract one or more health care associated infections (HAI), while approximately 20-30% of patients in critical care are affected.[12] This high incidence of HAI not only accounts for prolonged hospital stay and preventable morbidity and mortality of patients and additional costs, but also enlarges the global threat to human health due to the emergence of multi-resistant bacteria by using antibiotics needed to treat these infections.[13 14]

Adequate hand hygiene by all medical professionals has been recognized as an eminent measure to reduce transmission of (multi-resistant) pathogens.[14] However, adherence to hand hygiene guidelines, as with many quality improvement guidelines, is low, in particular among phycians [15] and medical students [16] Medical professionals' patient safety practices, including their (non)adherence to hand hygiene guidelines, should be traced back to how future medical professionals are trained today.[17-20] The inclusion of medical students in patient safety initiatives is vital, because students shape their behavior and form habits during their internships, making them a priority group to be targeted in interventions. [21]

In the last decades, research into the behavioural factors influencing the hand hygiene behaviour of health care professionals has received growing attention.[22 23] Assessment of hand hygiene behavioral factors has often been guided by behavioral theories, in particular the Theory of Planned Behavior (TPB), [24-28]. The TPB states that individual behaviour is influenced by attitudes, social norms (i.e. what people around you think and do) and self-efficacy (i.e. whether you feel that you are able to perform

For peer review only - http://bmjopen.bmj.com/site/about/guidelines.xhtml

the behavior).[29] This theory has been used to explain the behavior of physicians and nurses, but not yet that of medical students. Only a few studies into factors explaining hand hygiene compliance among medical students have been conducted so far, most lacking a theoretical behavioral framework or restricted to environmental factors such as access to facilities (low access associated with low compliance) and compliance of peers and superiors (low compliance of medical staff associated with low student compliance).[30 31] Van der Mortel et al.[16 32 33] investigated factors associated with the hand hygiene behavioural of students in Australia, Italy and Greece, showing that knowledge was regularly insufficient, differences in hand hygiene attitudes and behaviours between future doctors and nurses are already present at an undergraduate level and that better hand hygiene education is necessary. Therefore, to date knowledge is still rather limited on the behavioral factors that influence patient safety behaviors, including hand hygiene, of our next generation of physicians.[34] These insights can help clinical educators to promote patient safe behavior among medical students and thereby improve patient safety in the near future.

In this study we sought to identify correlates of the hand hygiene compliance of sixth-year medical students, using a theoretical behavioral framework.

Methods

Setting and participants

Over a period of 12 months we recruited a class of 322 medical interns; a researcher visited the class room during regular lessons and invited students to complete the paper and pencil questionnaire. All students were enrolled in their last compulsory internship (Public Health) at the Erasmus Medical Centre Rotterdam (a 1,320 bed university hospital). Every 2 weeks, a group of 10-15 students started their Public Health training and were invited to complete a questionnaire. Before this internship, the students followed an 18 month rotation schedule in all major specialties: internal medicine, surgery, pediatrics, gynaecology, neurology, psychiatry, ENT (Ear, Nose and Throat medicine), dermatology

For peer review only - http://bmjopen.bmj.com/site/about/guidelines.xhtml

and general practice. The interns rotated among the university medical centre and 20 non-academic hospitals within the region South-West Netherlands. These institutions serve over 6.3 million people of various social and ethnic backgrounds in a mixed urban and rural area. In the second year of the study, undergraduate medical students completed the compulsory two hour practical module ‘Basic Hygiene’ of the Unit Infection Prevention (Erasmus MC), in which they are taught the principles of hand hygiene, among other things.

A researcher or research assistant visited the class room during the lesson and invited all students to participate. Students only received a questionnaire if they indicated they were willing to participate. Questionnaires were completed anonymously and the researcher returned later to collect the completed questionnaires. This study is part of the hand hygiene project, which was provided a waiver by Institutional Review Board of Erasmus MC Rotterdam.

Patient and public involvement

Patients and the public were not involved in the design of the study, or in the recruitment to and conduct of the study.

Behavioural theory and questionnaire

We developed the questionnaire used in this study for a larger national study on the determinants of hand hygiene compliance. This questionnaire is based on a translated version of the Hand hygiene Assessment Instrument (HAI)[35] with additional constructs identified by qualitative research among physicians, nurses and medical students.[17] In particular, adequate knowledge of hand hygiene guidelines, hand hygiene as a habitual behaviour and risk perception (risk of contracting an infection yourself, and risk of patient contracting an infection) were identified in a qualitative study we performed. The HAI was translated into Dutch by two Dutch speakers and translated back into English by a native speaker to ensure the content had not changed. Experts in the field of behavioural science and infection control examined all items to ensure face and content validity.

For peer review only - http://bmjopen.bmj.com/site/about/guidelines.xhtml

The questionnaire is based on the TPB [29] , and a number of additional constructs from the Social Ecological Model (SEM) [36] and the Habit Scale Index [37] were added. **Figure 1** shows our extended TPB model. *Behaviour* (in this case self-reported compliance, a behavior in previous internships) is influenced by *intention* (whether you intend or plan to comply with guidelines), which in turn is influenced by *attitudes* (beliefs of the importance of hand hygiene; outcome beliefs, i.e. expected outcomes of hand hygiene); *social norms* (referent beliefs, i.e. beliefs about how other people think about hand hygiene; descriptive norm, i.e. the perceived behaviour of others) and *self efficacy* (the perception of whether students think they could perform hand hygiene). We added the following additional constructs: *knowledge of the guidelines*, *risk perception* (chance of infection occurring; severity of infection for self (i.e. student) or patient), and *habit* (hand hygiene is something you do without thinking about it), measured with the Self-report Index of Habit.[37] Since the internal consistency was at least adequate for each construct (Cronbachs $\alpha \geq .70$) we calculated average scores for use in further analysis (see **table 1**). All items, with the exception of the knowledge questions (measured by 5 true/false questions), were answered using 7 point Likert scales.
Self-reported compliance to hand hygiene guidelines, as outcome measure, was measured on a scale from 0 to 10 (never to always) for 13 potential hand hygiene situations (e.g. before touching a patient, before wound care, after patient contact).[35] For each respondent, we calculated an average score for use in further analysis.

Statistical analysis

We performed hierarchical multivariate linear regression analysis to identify the effects of the behavioural factors (independent variables) on self-reported compliance (dependent variable). The constructs were added to the model in 3 steps:

For peer review only - http://bmjopen.bmj.com/site/about/guidelines.xhtml

1) knowledge and risk perception,

2) all factors of step 1, with the addition of attitude, social norms and self-efficacy, and

3) all factors of step 2, with the addition of habit.

Comparison of self-reported compliance scores between male and female students was done by means of t-test for between group comparisons.

Results

Demographic data

In total, 313 (97%) students completed at least 75% of the questionnaire and were included in the analysis. The students had an average age of 25.3 years (Standard Deviation (SD) 2.9), and 201 (64%) of the students were female, which is representative of the male-female ratio of medical students in the Netherlands.[38]

Self-reported compliance

The average self-reported compliance was 8.1 on a 10 point scale (SD .96). This measure was 8.2 (SD .92) for females and 7.8 (SD .99) for males. This difference was statistically significant (P<.05). Self-reported compliance ranged from 4.3 (when resuming care after an interruption to 9.8 (after direct contact with body fluids).

Behavioural correlates

Table 2 shows the behavioural correlates associated with hand hygiene compliance in medical students. The regression coefficient β indicates the slope of the regression-line, and gives the average increase of compliance when the variable increases by 1. Knowledge of guidelines and risk perception explained 4.3% of the variance of self-reported compliance (adjusted R^2 = .043) (model 1). However, the contribution of these factors to self-reported hand hygiene compliance was not statistically significant.

For peer review only - http://bmjopen.bmj.com/site/about/guidelines.xhtml

For peer review only

The addition of attitude, social norms and self-efficacy (model 2), resulted in an explained variance of 27%: (R^2 =.270), with perceived outcomes (an element of attitude) (β=.231, *P*<.001) and self-efficacy (β=.306, *P*<.001) showing a statistically significant association with self-reported compliance.

In model 3, the addition of habit resulted in an explained variance of 40% (adjusted R^2=.401), with habit showing a strong and statistically significant association with self-reported compliance, (β=.471, *P*<.001). The associations of perceived outcomes and self-efficacy were somewhat weakened in this final model, but both remained statistically significant at the 5% level.

Discussion

The results of this study show that the hand hygiene behaviour of final year medical students, i.e. the new generation of physicians, is most strongly influenced by habit, perceived outcomes of hand hygiene and whether students feel they have the ability to perform hand hygiene in practice. Our extended behavioural model, which included attitudes, social norms, self-efficacy, knowledge, risk perception and habit, was able to explain a substantial part of the variance in self-reported compliance (adjusted R^2=.401).

One strength of our study is that we were able to include a full class of all medical students during one year, with a response of 97%. Students were approached to fill out the questionnaire after they had completed an 18 month rotation schedule of 9 specialties in both teaching and non-teaching facilities in a mixed urban/rural area. This class therefore had recently experienced a large number of hospital settings and patient types. After graduation, the students may select any clinical or non-clinical specialty, and our study population therefore represents juniors that will continue their career within a broad spectrum of medical disciplines. A second strength of this study is that we used a hand hygiene questionnaire, based on combined insights from the Theory of Planned Behaviour and Social Ecological Models.

For peer review only - http://bmjopen.bmj.com/site/about/guidelines.xhtml

The use of self-reported compliance as the primary outcome measure and lack of observational data form one of this study's limitations, and we are therefore only able to base our model on self-reported behaviour. In the setting of an internship in public health, where this study was conducted, opportunities for hand hygiene are almost absent and directly observing hand hygiene compliance in the multitude of very diverse medical institutions during the preceding rotation schedule would not have yielded comparable data. This rotation schedule also resulted in the use of a cross-sectional design, which restricts conclusions on causality. A longitudinal study would resolve this restriction but would be arduous due to the rotations and most likely result in a high loss due to follow up and a much lower response rate as a result. Therefore the use of self-reported data in a cross-sectional design was the best option in our case. Further, there is a possibility of social desirability bias since students completed the questionnaire in a class-room setting.

A second limitation of our study arises from our inability to explore the influence of cultural factors in our analyses. International patient safety experts have addressed the need to tackle not only individual change but also organizational change in order to improve patient safety culture.[1] A poor safety culture has been found to be associated with adverse events and a substantial improvement requires a culture of safety within the organization.[39] We would therefore have liked to include the construct culture in our model, but due to the large number of wards within different hospitals that students worked on (and therefore large variation in culture they might have experienced) we were unable to investigate its effects in this study. Culture could prove a valuable addition to the model and explain an additional part of the behaviour of medical students. The influence of culture should be further investigated in a different study design focusing on the observed compliance of interns of specific units, hospitals and/or specialties.

As a third limitation it must be mentioned that, although we used an extended version of the Theory of Planned Behavior, potential correlates of hand hygiene compliance could be omitted. Other

For peer review only - http://bmjopen.bmj.com/site/about/guidelines.xhtml

studies have used more comprehensive models, such as the Theory Domains Framework [40] and the Health Action Process Approach [41] to explain hand hygiene compliance of physicians and nurses and their application in medical students could be considered. Although the addition of the Habit Scale Index did add an extra 13% explained variance, indicating hand hygiene is a strongly habitual behavior, and interventions to improve it should not only focus on volitional construct

Only a few studies on the observed hand hygiene compliance of medical students have been conducted so far, mostly looking at other factors than student behavior (e.g. facilities), limiting a straightforward comparison of the results presented here [30 31]. We found that in addition to external factors such as access to facilities and compliance of superiors, student- related behavioural factors make their contribution as well. Previous research has shown that already at an undergraduate level difference between perception and knowledge towards hand hygiene can exist (with nurse more scoring more positively) [16], which we found also. A study from the UK found that the observed hand hygiene guideline compliance of medical students in an examinational setting was extremely low, even in the presence of 'Wash Your Hands' signs.[42] A hand hygiene intervention after the SARS (Severe acute respiratory syndrome) outbreak in Asia had good results, and was found to be related to a higher level of perceived risk; risk perception was not found to be significantly associated with hand hygiene compliance in this study, although this difference could be a result of the extreme situational circumstances during the SARS outbreak.[43]

Previously positive attitudes towards hand hygiene, and in particular positive beliefs about the outcome of performing hand hygiene have been found to be significantly associated with hand hygiene compliance of nurses and physicians.[23 44 45] , similar to the results we found here for medical students. The influence of habit [45] on hand hygiene behaviour had been understudied so far, and we found a strong association between habit and the self-reported hand hygiene behaviour of medical students. Since habit seems to play an important role in influencing hand hygiene behaviour, and the

For peer review only - http://bmjopen.bmj.com/site/about/guidelines.xhtml

foundation for these professional habits is laid down during medical training, it is important for the working environment of junior doctors to further stimulate strengthening these habits. Habit is a complex construct, referred to as a "habitual mind-set" in which people focus less on new information, but rather fall back on previously formed automatic cue-responses, thereby maintain that behaviour.[45] Actions which are repeated often enough in a stable context in order to shape a habitual mind-set. The behaviour then becomes automatic and could even overrule intentions.

Much of the behaviour of medical students is based on the behaviour of the role models (often residents) they encounter during their clinical phase, and not on what they have learned during their preclinical phase.[18 46 47] Once they reach their internship-phase, medical students are confronted with and adapt themselves to a culture of non-adherence. This effect is also present among residents, as with a senior member of the team performing hand hygiene, the hand hygiene compliance of residents increases significantly, but overall compliance of residents is as low as their qualified colleagues (< 40%).[46] It is therefore essential to break the vicious circle and one way to do this is by preparing medical students for the incongruences they will encounter in clinical practice and increase their coping skills. Based on habit theory, in the case of hand hygiene, it could be essential to shape the correct mind-set in the correct context (e.g. on the workfloor, as opposed to the class room) in order for strong habits to be formed.[45]

It is increasingly recognized that patient safety should be improved through education.[19 34] Medical students indicate themselves that more education on patient safety and especially hand hygiene is necessary.[48] Residents state that medical mistakes could be prevented with more education on the matter.[49] Our results show that traditional educational methods focusing on knowledge improvement are not the way to go in order to stimulate better patient safety behaviours, such as hand hygiene compliance. Similarly to physicians and nurses it is essential to target medical students with interventions tailored to the major modifiable determinants of non-compliance. Targeting

For peer review only

medical students' behaviour should focus on the empowerment of these juniors, rather than increasing their factual knowledge of procedures. Insights from the behavioural sciences may be useful to increase the self-efficacy of this important target group. Interventions using the concept of action planning or implementation intentions have been successful in several settings [50 51], including hospital care [52] and seem to be promising in this context.

Adequate hand hygiene can lead to a reduced rate of HAI and a drop in adverse events, morbidity and mortality. Application of behavioural insights can lead to patient safety improvements throughout health care, and ultimately to safer hospitals. Breaking through the culture of non-adherence is the first step to achieving this goal.

In conclusion, targeting medical students' behaviour should focus on the empowerment of these juniors and provide them with evidence on the health benefits of prevention, rather than increasing their factual knowledge of procedures. Clinical teaching environments could help them form good patient safety habits during this vital phase of their career.

For peer review only

For peer review only - http://bmjopen.bmj.com/site/about/guidelines.xhtml

Acknowledgments

The authors wish to thank medical students for their participation in this study.

Authors contribution

The study was conceived and designed by VE and EB. Data collection was performed by VE and RE and analyzed by VE.

VE, SO, ER, RE, MV, AB and EB were involved in the interpretation of the data. VE wrote the first draft of the manuscript. VE, SO, ER, RE, MV, AB and EB critically revised the manuscript. VE, SO, ER, RE, MV, AB and EB read and approved the final manuscript.

Funding

This work was supported by the Netherlands Organisation for Health Research and Development (*ZonMW*); grant number 50-50115-96-632.

Competing interests

The authors declare that they have no competing interests.

Patient consent

Not required.

Data sharing

No additional data are available.

For peer review only

References

1. Kohn LT, Corrigan JM, Donaldson MS, et al. *To err is human: Building a safer health system*. Washington D.C: National Academy Press, 2000.
2. Reason J. Human error: models and management. BMJ 2000;**320**(7237):768-70.
3. Helmreich RL. On error management: lessons from aviation. BMJ 2000;**320**(7237):781-5.
4. Wise J. MPs attack NHS for putting finances and targets ahead of patient safety. BMJ 2009;**339**.
5. Gawande AA, Thomas EJ, Zinner MJ, et al. The incidence and nature of surgical adverse events in Colorado and Utah in 1992. Surgery 1999;**126**(1):66-75.
6. Weiser TG, Makary MA, Haynes AB, et al. Standardised metrics for global surgical surveillance. Lancet 2009;**374**(9695):1113-7.
7. Zegers M, de Bruijne MC, de Keizer B, et al. The incidence, root-causes, and outcomes of adverse events in surgical units: implication for potential prevention strategies. Patient Saf Surg 2011;**5**:13.
8. Bates DW, Cullen DJ, Laird N, et al. Incidence of adverse drug events and potential adverse drug events. Implications for prevention. ADE Prevention Study Group. JAMA 1995;**274**(1):29-34.
9. Dequito AB, Mol PG, van Doormaal JE, et al. Preventable and non-preventable adverse drug events in hospitalized patients: a prospective chart review in the Netherlands. Drug Saf 2011;**34**(11):1089-100.
10. Burke JP. Infection control - a problem for patient safety. N Engl J Med 2003;**348**(7):651-6.
11. Pittet D, Donaldson L. Clean care is safer care: the first global challenge of the WHO World Alliance for Patient Safety. Am J Infect Control 2005;**33**(8):476-9.
12. World Health Organization. Chapter 3. The burden of endemic health care-associated infection in high-income countries. Report on the burden of endemic health care-associated infection worldwide. Geneva: WHO Press, 2011:12-15.
13. Donaldson L. Dirty hands: the human cost. CMO Annual Report 2006 2006.
14. World Health Organization. World Alliance for Patient Safety. The Global Patient Safety Challenge 2005–2006. Clean Care is Safer Care. Geneva, 2005.
15. Erasmus V, Daha TJ, Brug H, et al. Systematic review of studies on compliance with hand hygiene guidelines in hospital care. Infect Control Hosp Epidemiol 2010;**31**(3):283-94.
16. van de Mortel TF KS, Progano T, Sansoni J. A comparison of the hand hygiene knowledge, beliefs and practices of Italian nursing and medical students. J Adv Nurs 2012;**68**(3):569-79.
17. Erasmus V, Brouwer W, van Beeck EF, et al. A qualitative exploration of reasons for poor hand hygiene among hospital workers: lack of positive role models and of convincing evidence that hand hygiene prevents cross-infection. Infect Control Hosp Epidemiol 2009;**30**(5):415-9.
18. Lankford MG, Zembower TR, Trick WE, et al. Influence of role models and hospital design on hand hygiene of healthcare workers. Emerg Infect Dis 2003;**9**(2):217-23.
19. Paterson-Brown S. Improving patient safety through education. BMJ 2011;**342**:d214.
20. Snow M, White GL, Jr., Alder SC, et al. Mentor's hand hygiene practices influence student's hand hygiene rates. Am J Infect Control 2006;**34**(1):18-24.
21. Wakefield JG, McLaws ML, Whitby M, et al. Patient safety culture: factors that influence clinician involvement in patient safety behaviours. Qual Saf Health Care 2010;**19**(6):585-91.
22. Pittet D. Improving compliance with hand hygiene in hospitals. Infect Control Hosp Epidemiol 2000;**21**(6):381-6.
23. Whitby M, Pessoa-Silva CL, McLaws ML, et al. Behavioural considerations for hand hygiene practices: the basic building blocks. J Hosp Infect 2007;**65**(1):1-8.
24. O'Boyle CA, Henly SJ, Larson E. Understanding adherence to hand hygiene recommendations: the theory of planned behavior. Am J Infect Control 2001;**29**(6):352-60.
25. Jenner EA, Watson PW, Miller L, et al. Explaining hand hygiene practice: an extended application of the Theory of Planned Behaviour. Psychol Health Med 2002;**7**:311-26.

For peer review only - http://bmjopen.bmj.com/site/about/guidelines.xhtml

26. Sax H, Uckay I, Richet H, et al. Determinants of good adherence to hand hygiene among healthcare workers who have extensive exposure to hand hygiene campaigns. Infect Control Hosp Epidemiol 2007;**28**(11):1267-74.
27. De Wandel D, Maes L, Labeau S, et al. Behavioral determinants of hand hygiene compliance in intensive care units. Am J Crit Care 2010;**19**(3):230-9.
28. White KM, Jimmieson NL, Obst PL, et al. Using a theory of planned behaviour framework to explore hand hygiene beliefs at the '5 critical moments' among Australian hospital-based nurses. BMC Health Serv Res 2015;**15**:59.
29. Ajzen J. The theory of planned behavior. Organizational behavior and human decision processes 1991;**50**:179-211.
30. Hunt DC, Mohammudally A, Stone SP, et al. Hand-hygiene behaviour, attitudes and beliefs in first year clinical medical students. J Hosp Infect 2005;**59**(4):371-3.
31. Polacco MA, Shinkunas L, Perencevich EN, et al. See one, do one, teach one: hand hygiene attitudes among medical students, interns, and faculty. Am J Infect Control 2015;**43**(2):159-61.
32. van de Mortel TF, Apostolopoulou E, Petrikkos G. A comparison of the hand hygiene knowledge, beliefs, and practices of Greek nursing and medical students. Am J Infect Control 2010;**38**(1):75-7.
33. van de Mortel TF. Development of a questionnaire to assess health care students' hand hygiene knowledge, beliefs and practices. Aust J Adv Nurs 2009;**26**(3):9-16.
34. Humphreys H, Richards J. Undergraduate and postgraduate medical education on the prevention and control of healthcare-associated infection. Int J Infect Control 2011; **v7**::i2.
35. O'Boyle C A, Henly SJ, Duckett LJ. Nurses' motivation to wash their hands: a standardized measurement approach. Appl Nurs Res 2001;**14**(3):136-45.
36. Bronfenbrenner U. *The ecology of human development*. Cambridge, MA: Harvard University Press, 1979.
37. Verplanken B, Orbell S. Reflections on Past Behavior: A Self-Report Index of Habit Strength. Journal of Applied Social Psychology;**33**(6):1313-30.
38. van der Velden LF, Hingstman L, Heiligers PJ, et al. [Increasing number of women in medicine: past, present and future] Toenemend percentage vrouwen in de geneeskunde: verleden, heden en toekomst. Ned Tijdschr Geneeskd 2008;**152**(40):2165-71.
39. Nieva VF, Sorra J. Safety culture assessment: a tool for improving patient safety in healthcare organizations. Qual Saf Health Care 2003;**12 Suppl 2**:ii17-23.
40. Fuller C, Besser S, Savage J, et al. Application of a theoretical framework for behavior change to hospital workers' real-time explanations for noncompliance with hand hygiene guidelines. Am J Infect Control 2014;**42**(2):106-10.
41. von Lengerke T, Lutze B, Graf K, et al. Psychosocial determinants of self-reported hand hygiene behaviour: a survey comparing physicians and nurses in intensive care units. J Hosp Infect 2015;**91**(1):59-67.
42. Feather A, Stone SP, Wessier A, et al. 'Now please wash your hands': the handwashing behaviour of final MBBS candidates. Journal of Hospital Infection 2000;**45**(1):62-64.
43. Wong TW, Tam WW. Handwashing practice and the use of personal protective equipment among medical students after the SARS epidemic in Hong Kong. Am J Infect Control 2005;**33**(10):580-6.
44. Pittet D, Simon A, Hugonnet S, et al. Hand hygiene among physicians: performance, beliefs, and perceptions. Ann Intern Med 2004;**141**(1):1-8.
45. Verplanken B, Aarts H. Habit, Attitude and Planned Behaviour: Is Habit an Empty Construct or an Interesting Case of Goal-Directed Automaticity? Eur Rev Soc Psychol 1999;**10**:101-34.
46. Rome M, Sabel A, Price CS, et al. Hand hygiene compliance. J Hosp Infect 2007;65(2):173.
47. Walton MM. Hierarchies: the Berlin Wall of patient safety. Qual Saf Health Care 2006;**15**(4):229-30.

For peer review only - http://bmjopen.bmj.com/site/about/guidelines.xhtml

48. Mann CM, Wood A. How much do medical students know about infection control? J Hosp Infect 2006;**64**(4):366-70.
49. Varkey P, Karlapudi S, Rose S, et al. A patient safety curriculum for graduate medical education: results from a needs assessment of educators and patient safety experts. Am J Med Qual 2009;**24**(3):214-21.
50. Gollwitzer PM, Sheeran P. Implementation intentions and goal achievement: a meta-analysis of effects and processes. Adv Exp Soc Psychol 2006;**38**:69-119.
51. Sniehotta FF, Scholz U, Schwarzer R. Bridging the intention-behaviour gap: Planning, self-efficacy, and action control in the adoption and maintenance of physical exercise. Psychol Health 2005;**20**(2):143-60.
52. Erasmus V, Kuperus MN, Richardus JH, et al. Improving hand hygiene behaviour of nurses using action planning: a pilot study in the intensive care unit and surgical ward. J Hosp Infect 2010;**76**(2):161-4.

For peer review only

Table 1: Constructs of the questionnaire on behavioural determinants of hand hygiene with example questions and internal consistency (Cronbach's α)

Construct	# items	Mean (SD)	Cronbach's α	Example
**Knowledge**	5	4.3 (.78)	-	5 true/false questions about factual knowledge
**Risk perception**:				
Chance	3	5.6 (1,7)	0.76	How big is the chance that an infection will occur
Severity self	1	5.6 (2.3)	-	How severe will the consequences of an infection be for myself
Severity patient	1	7.2 (1.5)	-	How severe will the consequences of an infection be for my patient
**Attitudes**:				
Beliefs about hand hygiene	8	5.3 (.81)	0.76	Hand hygiene is something I find important
Perceived outcomes	5	5.0 (.98)	0.78	If I follow that hand hygiene guidelines my patients will develop fewer infections
**Social norms**:				
Referent beliefs	4	4.7 (1.2)	0.91	My superior thinks that I should always follow the hand hygiene guidelines
Descriptive norm	8	3.5 (.81)	0.73	My colleagues always follow the hand hygiene guidelines
**Self-efficacy**	11	4.8 (.94)	0.89	I am certain that I will be able to follow the hand hygiene guidelines
**Habit**	12	4.7 (1.1)	0.95	Following the hand hygiene guidelines is something I do without thinking about it

For peer review only

Table 2: Behavioural correlates of hand hygiene compliance of medical students (n=313)

	Model 1		Model 2			Model 3		
	β	R^2	B	R^2	$R^{2change}$	β	R^2	$R^{2change}$
		.043		.270	.189		.401	.131
Knowledge	.081		.044			.063		
Risk perception:								
Chance	.101		.049			.010		
Severity self	.105		.041			.002		
Severity patient	.102		.036			.019		
Attitude:								
Beliefs			.103			-.026		
Perceived outcomes			.231***			.174**		
Social norms:								
Referent beliefs			.003			-.001		
Descriptive norm			.063			.037		
Self efficacy			.306***			.138*		
Habit						.471***		

* P<.05; ** P<.01; *** P < .001

Model 1: knowledge + risk perception

Model 2: knowledge + risk perception + attitudes + social norms + self efficacy

Model 3: knowledge + risk perception + attitudes + social norms + self efficacy + habit

For peer review only

Figure 1: Extended Theory of Planned Behavior model. Constructs in gray boxes have been added.

For peer review only

For peer review only - http://bmjopen.bmj.com/site/about/guidelines.xhtml

Figure

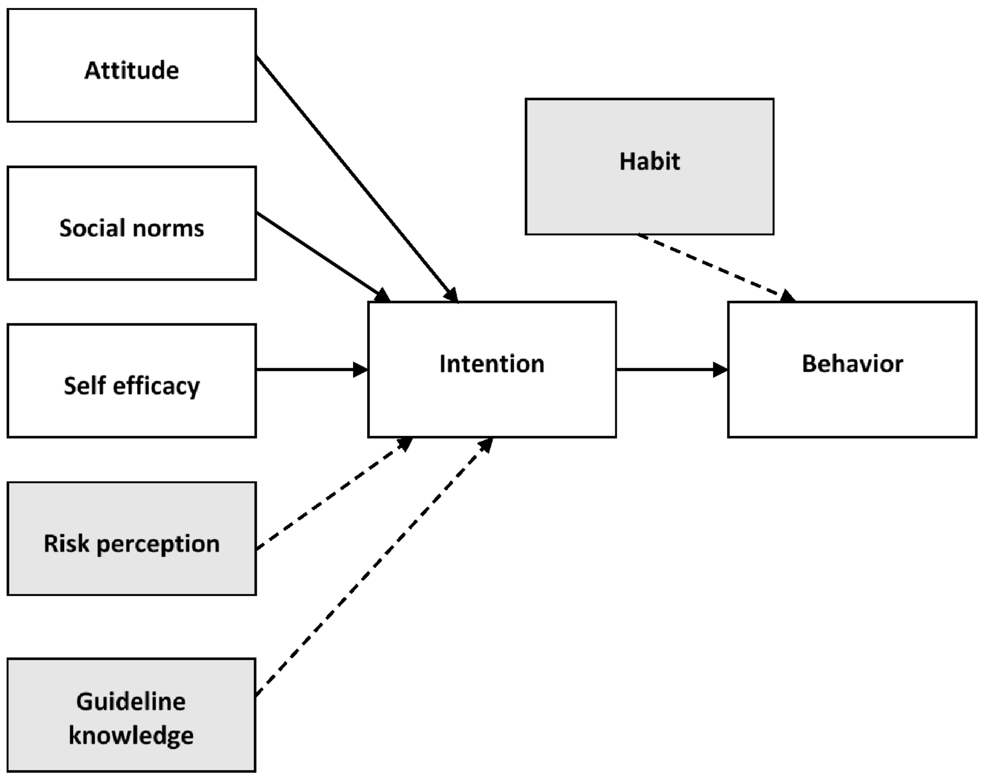
Attitude
Social norms
Self efficacy
Risk perception
Guideline knowledge
Habit
Intention
Behavior

Reporting checklist for cross sectional study.

Based on the STROBE cross sectional guidelines.

Instructions to authors

Complete this checklist by entering the page numbers from your manuscript where readers will find each of the items listed below.

Your article may not currently address all the items on the checklist. Please modify your text to include the missing information. If you are certain that an item does not apply, please write "n/a" and provide a short explanation.

Upload your completed checklist as an extra file when you submit to a journal.

In your methods section, say that you used the STROBE cross sectional reporting guidelines, and cite them as:

von Elm E, Altman DG, Egger M, Pocock SJ, Gotzsche PC, Vandenbroucke JP. The Strengthening the Reporting of Observational Studies in Epidemiology (STROBE) Statement: guidelines for reporting observational studies.

		Reporting Item	Page Number
Title	#1a	Indicate the study’s design with a commonly used term in the title or the abstract	1
Abstract	#1b	Provide in the abstract an informative and balanced summary of what was done and what was found	2
Background / rationale	#2	Explain the scientific background and rationale for the investigation being reported	4
Objectives	#3	State specific objectives, including any prespecified hypotheses	5
Study design	#4	Present key elements of study design early in the paper	5
Setting	#5	Describe the setting, locations, and relevant dates, including periods of recruitment, exposure, follow-up, and data collection	5
Eligibility criteria	#6a	Give the eligibility criteria, and the sources and methods of selection of participants.	5

For peer review only

	#7	Clearly define all outcomes, exposures, predictors, potential confounders, and effect modifiers. Give diagnostic criteria, if applicable	6
Data sources / measurement	#8	For each variable of interest give sources of data and details of methods of assessment (measurement). Describe comparability of assessment methods if there is more than one group. Give information separately for for exposed and unexposed groups if applicable.	5-6
Bias	#9	Describe any efforts to address potential sources of bias	n/a
Study size	#10	Explain how the study size was arrived at	5
Quantitative variables	#11	Explain how quantitative variables were handled in the analyses. If applicable, describe which groupings were chosen, and why	7
Statistical methods	#12a	Describe all statistical methods, including those used to control for confounding	7
	#12b	Describe any methods used to examine subgroups and interactions	n/a
	#12c	Explain how missing data were addressed	n/a
	#12d	If applicable, describe analytical methods taking account of sampling strategy	n/a
	#12e	Describe any sensitivity analyses	n/a
Participants	#13a	Report numbers of individuals at each stage of study—eg numbers potentially eligible, examined for eligibility, confirmed eligible, included in the study, completing follow-up, and analysed. Give information separately for for exposed and unexposed groups if applicable.	8
	#13b	Give reasons for non-participation at each stage	n/a
	#13c	Consider use of a flow diagram	n/a
Descriptive data	#14a	Give characteristics of study participants (eg demographic, clinical, social) and information on exposures and potential confounders. Give information separately for exposed and unexposed groups if applicable.	8

For peer review only

	#14b	Indicate number of participants with missing data for each variable of interest	n/a
Outcome data	#15	Report numbers of outcome events or summary measures. Give information separately for exposed and unexposed groups if applicable.	8-9, 19
Main results	#16a	Give unadjusted estimates and, if applicable, confounder-adjusted estimates and their precision (eg, 95% confidence interval). Make clear which confounders were adjusted for and why they were included	n/a
	#16b	Report category boundaries when continuous variables were categorized	n/a
	#16c	If relevant, consider translating estimates of relative risk into absolute risk for a meaningful time period	n/a
Other analyses	#17	Report other analyses done—e.g., analyses of subgroups and interactions, and sensitivity analyses	n/a
Key results	#18	Summarise key results with reference to study objectives	8
Limitations	#19	Discuss limitations of the study, taking into account sources of potential bias or imprecision. Discuss both direction and magnitude of any potential bias.	9-10
Interpretation	#20	Give a cautious overall interpretation considering objectives, limitations, multiplicity of analyses, results from similar studies, and other relevant evidence.	13
Generalisability	#21	Discuss the generalisability (external validity) of the study results	11-12
Funding	#22	Give the source of funding and the role of the funders for the present study and, if applicable, for the original study on which the present article is based	14

The STROBE checklist is distributed under the terms of the Creative Commons Attribution License CC-BY. This checklist was completed on 12. December 2018 using http://www.goodreports.org/, a tool made by the EQUATOR Network in collaboration with Penelope.ai

For peer review only

BMJ Open

Assessment of correlates of hand hygiene compliance among final year medical students: a cross-sectional study in the Netherlands

Journal:	*BMJ Open*
Manuscript ID	bmjopen-2019-029484.R2
Article Type:	Original research
Date Submitted by the Author:	17-Dec-2019
Complete List of Authors:	Erasmus, Vicki; Erasmus MC, Public Health<br>Otto, Suzie; Erasmus MC, Public Health<br>De Roos, E; Erasmus MC, Public Health<br>van Eijsden, Rianne; Erasmus MC, Public Health<br>Vos, Margreet; Erasmus MC Medical Center Rotterdam, Department of Medical Microbiology and Infectious diseases<br>Burdorf, Alex; Erasmus MC, Department of Public Health<br>VAN BEECK, Ed ; Erasmus MC, Public Health
**Primary Subject Heading**:	Medical education and training
Secondary Subject Heading:	Public health
Keywords:	hand hygiene, compliance, behavior, habit, medical students

SCHOLARONE™
Manuscripts

I, the Submitting Author has the right to grant and does grant on behalf of all authors of the Work (as defined in the below author licence), an exclusive licence and/or a non-exclusive licence for contributions from authors who are: i) UK Crown employees; ii) where BMJ has agreed a CC-BY licence shall apply, and/or iii) in accordance with the terms applicable for US Federal Government officers or employees acting as part of their official duties; on a worldwide, perpetual, irrevocable, royalty-free basis to BMJ Publishing Group Ltd ("BMJ") its licensees and where the relevant Journal is co-owned by BMJ to the co-owners of the Journal, to publish the Work in this journal and any other BMJ products and to exploit all rights, as set out in our licence.

The Submitting Author accepts and understands that any supply made under these terms is made by BMJ to the Submitting Author unless you are acting as an employee on behalf of your employer or a postgraduate student of an affiliated institution which is paying any applicable article publishing charge ("APC") for Open Access articles. Where the Submitting Author wishes to make the Work available on an Open Access basis (and intends to pay the relevant APC), the terms of reuse of such Open Access shall be governed by a Creative Commons licence – details of these licences and which Creative Commons licence will apply to this Work are set out in our licence referred to above.

Other than as permitted in any relevant BMJ Author's Self Archiving Policies, I confirm this Work has not been accepted for publication elsewhere, is not being considered for publication elsewhere and does not duplicate material already published. I confirm all authors consent to publication of this Work and authorise the granting of this licence.

For peer review only

Assessment of correlates of hand hygiene compliance among final year medical students: a cross-sectional study in the Netherlands

Vicki Erasmus [1], Suzie J Otto [1], Emmely W. de Roos [1], Rianne van Eijsden [1], Margreet C Vos [2], Alex Burdorf [1], Ed F van Beeck [1]

[1] Department of Public Health, University Medical Center Rotterdam, 3000 CA Rotterdam, the Netherlands

[2] Department of Medical Microbiology and Infectious Diseases, University Medical Center Rotterdam, 3000 CA, Rotterdam, the Netherlands

Correspondence should be addressed to:

Vicki Erasmus

Erasmus University Medical Center

Department of Public Health

P.O. Box 2040

3000 CA Rotterdam

The Netherlands

Tel: +31 10 70 38777

v.erasmus@erasmusmc.nl

Key words: hand hygiene, compliance, behaviour, habit, medical students

Word count: 3 038

For peer review only

Abstract

Objectives To identify the factors that influence the hand hygiene compliance of final year medical students, using a theoretical behavioural framework.

Design Cross-sectional survey assessing self-reported compliance and its behavioural correlates.

Setting

Internships of medical students in the Netherlands.

Participants

322 Medical students of the Erasmus Medical Center were recruited over a period of 12 months during the Public Health internship, which is the final compulsory internship after an 18 month rotation schedule in all major specialties.

Primary and secondary outcome measures

Behavioural factors influencing compliance to hand hygiene guidelines were measured by means of a questionnaire based on the Theory of Planned Behaviour and Social Ecological Models. Multiple linear regression analysis was used to identify the effect of including attitudes, social norms, self-efficacy, knowledge, risk perception and habit on hand hygiene compliance.

Results

We included 313 students in the analysis (response rate 97%). The behavioural model explained 40% of the variance in self-reported compliance (adjusted R2 = 0.40). Hand hygiene compliance was strongly influenced by attitudes (perceived outcomes of preventive actions), self-efficacy (perception of the ability to perform hand hygiene at the clinical ward) and habit, but was not associated with knowledge and risk perception.

Conclusions

Targeting medical students' behaviour should focus on the empowerment of these juniors and provide them with evidence on the health benefits of prevention, rather than increasing their factual knowledge of procedures. Clinical teaching environments could help them form good patient safety habits during this vital phase of their career.

For peer review only - http://bmjopen.bmj.com/site/about/guidelines.xhtml

Article Summary

Strengths and limitations of this study

1. This study uses a hand hygiene questionnaire, based on insights from both the Theory of Planned Behaviour and Social Ecological Models.
2. The hand hygiene behaviour of medical students is investigated from a behavioural perspective in a large sample of students at the same stage of their internships.
3. The main limitations of this study are its cross-sectional design and the self-reported compliance with hand hygiene guidelines.

For peer review only

For peer review only - http://bmjopen.bmj.com/site/about/guidelines.xhtml

Introduction

Patient safety made its entrance into the fields of medical research and practice in the last decade of the twentieth century.[1-3] Ever since, patient safety has become a rising priority among health institutions, governments and insurance companies, who are all seeking to reduce the human and financial costs of preventable adverse events.[4] Prioritizing these events based on their impact and frequency of occurrence, shows that - next to surgical [5-7] and medication procedures [8 9] - infection prevention is a key element to improve patient safety.[10 11] In high income countries, 3.5% to 12% of all hospitalized patients contract one or more health care associated infections (HAI), while approximately 20-30% of patients in critical care are affected.[12] This high incidence of HAI not only accounts for prolonged hospital stay and preventable morbidity and mortality of patients and additional costs, but also enlarges the global threat to human health due to the emergence of multi-resistant bacteria by using antibiotics needed to treat these infections.[13 14]

Adequate hand hygiene by all medical professionals has been recognized as an eminent measure to reduce transmission of (multi-resistant) pathogens.[14] However, adherence to hand hygiene guidelines, as with many quality improvement guidelines, is low, in particular among physicians [15] and medical students [16] Medical professionals' patient safety practices, including their (non)adherence to hand hygiene guidelines, should be traced back to how future medical professionals are trained today.[17-20] The inclusion of medical students in patient safety initiatives is vital, because students shape their behaviour and form habits during their internships, making them a priority group to be targeted in interventions. [21]

In the last decades, research into the behavioural factors influencing the hand hygiene behaviour of health care professionals has received growing attention.[22 23] Assessment of hand hygiene behavioural factors has often been guided by behavioural theories, in particular the Theory of Planned Behaviour (TPB), [24-28]. The TPB states that individual behaviour is influenced by attitudes, social norms (i.e. what people around you think and do) and self-efficacy (i.e. whether you feel that

For peer review only - http://bmjopen.bmj.com/site/about/guidelines.xhtml

you are able to perform the behaviour).[29] This theory has been used to explain the behaviour of physicians and nurses, but not yet that of medical students. Only a few studies into factors explaining hand hygiene compliance among medical students have been conducted so far, most lacking a theoretical behavioural framework or restricted to environmental factors such as access to facilities (low access associated with low compliance) and compliance of peers and superiors (low compliance of medical staff associated with low student compliance).[30 31] Van de Mortel et al. [16 32 33] investigated factors associated with the hand hygiene behavioural of students in Australia, Italy and Greece, showing that knowledge was regularly insufficient, differences in hand hygiene attitudes and behaviours between future doctors and nurses are already present at an undergraduate level and that better hand hygiene education is necessary. Therefore, to date knowledge is still rather limited on the behavioural factors that influence patient safety behaviours, including hand hygiene, of our next generation of physicians.[34] These insights can help clinical educators to promote patient safe behaviour among medical students and thereby improve patient safety in the near future.

In this study we sought to identify correlates of the hand hygiene compliance of sixth-year medical students, using a theoretical behavioural framework.

Methods

Setting and participants

Over a period of 12 months we recruited a class of 322 medical interns; a researcher visited the class room during regular lessons and invited students to complete the paper and pencil questionnaire. All students were enrolled in their last compulsory internship (Public Health) at the Erasmus Medical Centre Rotterdam (a 1,320 bed university hospital). Every 2 weeks, a group of 10-15 students started their Public Health training and were invited to complete a questionnaire. Before this internship, the students followed an 18 month rotation schedule in all major specialties: internal medicine, surgery, pediatrics, gynaecology, neurology, psychiatry, ENT (Ear, Nose and Throat medicine), dermatology

For peer review only - http://bmjopen.bmj.com/site/about/guidelines.xhtml

and general practice. The interns rotated among the university medical centre and 20 non-academic hospitals within the region South-West Netherlands. These institutions serve over 6.3 million people of various social and ethnic backgrounds in a mixed urban and rural area. In the second year of the study, undergraduate medical students completed the compulsory two hour practical module 'Basic Hygiene' of the Unit Infection Prevention (Erasmus MC), in which they are taught the principles of hand hygiene, among other things.

A researcher or research assistant visited the class room during the lesson and invited all students to participate. Students only received a questionnaire if they indicated they were willing to participate. Questionnaires were completed anonymously and the researcher returned later to collect the completed questionnaires. This study is part of the hand hygiene project, which was provided a waiver by Institutional Review Board of Erasmus MC Rotterdam.

For peer review only

Patient and public involvement

Patients and the public were not involved in the design of the study, or in the recruitment to and conduct of the study.

Behavioural theory and questionnaire

We developed the questionnaire used in this study for a larger national study on the determinants of hand hygiene compliance. This questionnaire is based on a translated version of the Hand hygiene Assessment Instrument (HAI) [35] with additional constructs identified by qualitative research among physicians, nurses and medical students.[17] In particular, adequate knowledge of hand hygiene guidelines, hand hygiene as a habitual behaviour and risk perception (risk of contracting an infection yourself, and risk of patient contracting an infection) were identified in a qualitative study we performed. The HAI was translated into Dutch by two Dutch speakers and translated back into English by a native speaker to ensure the content had not changed. Experts in the field of

For peer review only - http://bmjopen.bmj.com/site/about/guidelines.xhtml

behavioural science (n=3) and infection control (n=3) examined all items to ensure face and content validity.

The questionnaire is based on the TPB [29] , and a number of additional constructs from the Social Ecological Model (SEM) [36] and the Habit Scale Index [37] were added. **Figure 1** shows our extended TPB model. *Behaviour* (in this case self-reported compliance, a behaviour in previous internships) is influenced by *intention* (whether you intend or plan to comply with guidelines), which in turn is influenced by *attitudes* (beliefs of the importance of hand hygiene; outcome beliefs, i.e. expected outcomes of hand hygiene); *social norms* (referent beliefs, i.e. beliefs about how other people think about hand hygiene; descriptive norm, i.e. the perceived behaviour of others) and *self efficacy* (the perception of whether students think they could perform hand hygiene). We added the following additional constructs: *knowledge of the guidelines*, *risk perception* (chance of infection occurring; severity of infection for self (i.e. student) or patient), and *habit* (hand hygiene is something you do without thinking about it), measured with the Self-report Index of Habit.[37] Since the internal consistency was at least adequate for each construct (Cronbach's $\alpha \geq .70$) we calculated average scores for use in further analysis (see **table 1**). All items, with the exception of the knowledge questions (measured by 5 true/false questions), were answered using 7 point Likert scales.

Self-reported compliance to hand hygiene guidelines, as outcome measure, was measured on a scale from 0 to 10 (never to always) for 13 potential hand hygiene situations (e.g. before touching a patient, before wound care, after patient contact).[35] For each respondent, we calculated an average score for use in further analysis.

Statistical analysis

We performed hierarchical multivariate linear regression analysis to identify the effects of the behavioural factors (independent variables) on self-reported compliance (dependent variable). The constructs were added to the model in 3 steps:

For peer review only - http://bmjopen.bmj.com/site/about/guidelines.xhtml

1) knowledge and risk perception,

2) all factors of step 1, with the addition of attitude, social norms and self-efficacy, and

3) all factors of step 2, with the addition of habit.

Comparison of self-reported compliance scores between male and female students was done by means of t-test for between group comparisons.

Results

Demographic data

In total, 313 (97%) students completed at least 75% of the questionnaire and were included in the analysis. The students had an average age of 25.3 years (Standard Deviation (SD) 2.9), and 201 (64%) of the students were female, which is representative of the male-female ratio of medical students in the Netherlands.[38]

Self-reported compliance

The average self-reported compliance was 8.1 on a 10 point scale (SD .96). This measure was 8.2 (SD .92) for females and 7.8 (SD .99) for males. This difference was statistically significant (P<.05). Self-reported compliance ranged from 4.3 (when resuming care after an interruption to 9.8 (after direct contact with body fluids).

Behavioural correlates

Table 2 shows the behavioural correlates associated with hand hygiene compliance in medical students. The regression coefficient β indicates the slope of the regression-line, and gives the average increase of compliance when the variable increases by 1. Knowledge of guidelines and risk perception explained 4.3% of the variance of self-reported compliance (adjusted R^2 = .043) (model 1). However, the contribution of these factors to self-reported hand hygiene compliance was not statistically significant.

For peer review only - http://bmjopen.bmj.com/site/about/guidelines.xhtml

For peer review only

The addition of attitude, social norms and self-efficacy (model 2), resulted in an explained variance of 27%: (R^2 =.270), with perceived outcomes (an element of attitude) (β=.231, *P*<.001) and self-efficacy (β=.306, *P*<.001) showing a statistically significant association with self-reported compliance.

In model 3, the addition of habit resulted in an explained variance of 40% (adjusted R^2=.401), with habit showing a strong and statistically significant association with self-reported compliance, (β=.471, *P*<.001). The associations of perceived outcomes and self-efficacy were somewhat weakened in this final model, but both remained statistically significant at the 5% level.

For peer review only

Discussion

The results of this study show that the hand hygiene behaviour of final year medical students, i.e. the new generation of physicians, is most strongly influenced by habit, perceived outcomes of hand hygiene and whether students feel they have the ability to perform hand hygiene in practice. Our extended behavioural model, which included attitudes, social norms, self-efficacy, knowledge, risk perception and habit, was able to explain a substantial part of the variance in self-reported compliance (adjusted R^2=.401).

One strength of our study is that we were able to include a full class of all medical students during one year, with a response of 97%. Students were approached to fill out the questionnaire after they had completed an 18 month rotation schedule of 9 specialties in both teaching and non-teaching facilities in a mixed urban/rural area. This class therefore had recently experienced a large number of hospital settings and patient types. After graduation, the students may select any clinical or non-clinical specialty, and our study population therefore represents juniors that will continue their career within a broad spectrum of medical disciplines. A second strength of this study is that we used a hand hygiene questionnaire, based on combined insights from the Theory of Planned Behaviour and Social Ecological Models.

For peer review only - http://bmjopen.bmj.com/site/about/guidelines.xhtml

The use of self-reported compliance as the primary outcome measure and lack of observational data form one of this study's limitations, and we are therefore only able to base our model on self-reported behaviour. In the setting of an internship in public health, where this study was conducted, opportunities for hand hygiene are almost absent and directly observing hand hygiene compliance in the multitude of very diverse medical institutions during the preceding rotation schedule would not have yielded comparable data. This rotation schedule also resulted in the use of a cross-sectional design, which restricts conclusions on causality. A longitudinal study would resolve this restriction but would be arduous due to the rotations and most likely result in a high loss due to follow up and a much lower response rate as a result. Therefore the use of self-reported data in a cross-sectional design was the best option in our case. Further, there is a possibility of social desirability bias since students completed the questionnaire in a class-room setting.[39]

A second limitation of our study arises from our inability to explore the influence of cultural factors in our analyses. International patient safety experts have addressed the need to tackle not only individual change but also organizational change in order to improve patient safety culture.[1] A poor safety culture has been found to be associated with adverse events and a substantial improvement requires a culture of safety within the organization.[40] We would therefore have liked to include the construct culture in our model, but due to the large number of wards within different hospitals that students worked on (and therefore large variation in culture they might have experienced) we were unable to investigate its effects in this study. Culture could prove a valuable addition to the model and explain an additional part of the behaviour of medical students. The influence of culture should be further investigated in a different study design focusing on the observed compliance of interns of specific units, hospitals and/or specialties.

As a third limitation it must be mentioned that, although we used an extended version of the Theory of Planned Behaviour, potential correlates of hand hygiene compliance could be omitted. Other

For peer review only - http://bmjopen.bmj.com/site/about/guidelines.xhtml

studies have used more comprehensive models, such as the Theory Domains Framework [41] and the Health Action Process Approach [42] to explain hand hygiene compliance of physicians and nurses and their application in medical students could be considered. Although the addition of the Habit Scale Index did add an extra 13% explained variance, indicating hand hygiene is a strongly habitual behaviour, and interventions to improve it should not only focus on volitional construct

Only a few studies on the observed hand hygiene compliance of medical students have been conducted so far, mostly looking at other factors than student behaviour (e.g. facilities), limiting a straightforward comparison of the results presented here [30 31]. We found that in addition to external factors such as access to facilities and compliance of superiors, student- related behavioural factors make their contribution as well. Previous research has shown that already at an undergraduate level difference between perception and knowledge towards hand hygiene can exist (with nurse more scoring more positively) [16], which we found also. A study from the UK found that the observed hand hygiene guideline compliance of medical students in an examinational setting was extremely low, even in the presence of 'Wash Your Hands' signs.[43] A hand hygiene intervention after the SARS (Severe acute respiratory syndrome) outbreak in Asia had good results, and was found to be related to a higher level of perceived risk; risk perception was not found to be significantly associated with hand hygiene compliance in this study, although this difference could be a result of the extreme situational circumstances during the SARS outbreak.[44]

Previously positive attitudes towards hand hygiene, and in particular positive beliefs about the outcome of performing hand hygiene have been found to be significantly associated with hand hygiene compliance of nurses and physicians.[23 45 46] , similar to the results we found here for medical students. The influence of habit [46] on hand hygiene behaviour had been understudied so far, and we found a strong association between habit and the self-reported hand hygiene behaviour of medical students. Since habit seems to play an important role in influencing hand hygiene behaviour, and the

For peer review only - http://bmjopen.bmj.com/site/about/guidelines.xhtml

foundation for these professional habits is laid down during medical training, it is important for the working environment of junior doctors to further stimulate strengthening these habits. Habit is a complex construct, referred to as a "habitual mind-set" in which people focus less on new information, but rather fall back on previously formed automatic cue-responses, thereby maintain that behaviour.[46] Actions which are repeated often enough in a stable context in order to shape a habitual mind-set. The behaviour then becomes automatic and could even overrule intentions.

Much of the behaviour of medical students is based on the behaviour of the role models (often residents) they encounter during their clinical phase, and not on what they have learned during their preclinical phase.[18 47 48] Once they reach their internship-phase, medical students are confronted with and adapt themselves to a culture of non-adherence. This effect is also present among residents, as with a senior member of the team performing hand hygiene, the hand hygiene compliance of residents increases significantly, but overall compliance of residents is as low as their qualified colleagues (< 40%).[47] It is therefore essential to break the vicious circle and one way to do this is by preparing medical students for the incongruences they will encounter in clinical practice and increase their coping skills. Based on habit theory, in the case of hand hygiene, it could be essential to shape the correct mind-set in the correct context (e.g. on the workfloor, as opposed to the class room) in order for strong habits to be formed.[46]

It is increasingly recognized that patient safety should be improved through education.[19 34] Medical students indicate themselves that more education on patient safety and especially hand hygiene is necessary.[49] Residents state that medical mistakes could be prevented with more education on the matter.[50] Our results show that traditional educational methods focusing on knowledge improvement are not the way to go in order to stimulate better patient safety behaviours, such as hand hygiene compliance. Similarly to physicians and nurses it is essential to target medical students with interventions tailored to the major modifiable determinants of non-compliance. Targeting

For peer review only - http://bmjopen.bmj.com/site/about/guidelines.xhtml

medical students' behaviour should focus on the empowerment of these juniors, rather than increasing their factual knowledge of procedures. Insights from the behavioural sciences may be useful to increase the self-efficacy of this important target group. Interventions using the concept of action planning or implementation intentions have been successful in several settings [51 52], including hospital care [53] and seem to be promising in this context.

Adequate hand hygiene can lead to a reduced rate of HAI and a drop in adverse events, morbidity and mortality. Application of behavioural insights can lead to patient safety improvements throughout health care, and ultimately to safer hospitals. Breaking through the culture of non-adherence is the first step to achieving this goal.

In conclusion, targeting medical students' behaviour should focus on the empowerment of these juniors and provide them with evidence on the health benefits of prevention, rather than increasing their factual knowledge of procedures. Clinical teaching environments could help them form good patient safety habits during this vital phase of their career.

For peer review only

For peer review only - http://bmjopen.bmj.com/site/about/guidelines.xhtml

Acknowledgments

The authors wish to thank medical students for their participation in this study.

Authors contribution

The study was conceived and designed by VE and EB. Data collection was performed by VE and RE and analyzed by VE.

VE, SO, ER, RE, MV, AB and EB were involved in the interpretation of the data. VE wrote the first draft of the manuscript. VE, SO, ER, RE, MV, AB and EB critically revised the manuscript. VE, SO, ER, RE, MV, AB and EB read and approved the final manuscript.

Funding

This work was supported by the Netherlands Organisation for Health Research and Development (*ZonMW*); grant number 50-50115-96-632.

Competing interests

The authors declare that they have no competing interests.

Patient consent

Not required.

Data availability statement

All data relevant to the study are included in the article or uploaded as supplementary information. No additional data are available.

For peer review only

References

1. Kohn LT, Corrigan JM, Donaldson MS, et al. *To err is human: Building a safer health system*. Washington D.C: National Academy Press, 2000.
2. Reason J. Human error: models and management. BMJ 2000;**320**(7237):768-70.
3. Helmreich RL. On error management: lessons from aviation. BMJ 2000;**320**(7237):781-5.
4. Wise J. MPs attack NHS for putting finances and targets ahead of patient safety. BMJ 2009;**339**.
5. Gawande AA, Thomas EJ, Zinner MJ, et al. The incidence and nature of surgical adverse events in Colorado and Utah in 1992. Surgery 1999;**126**(1):66-75.
6. Weiser TG, Makary MA, Haynes AB, et al. Standardised metrics for global surgical surveillance. Lancet 2009;**374**(9695):1113-7.
7. Zegers M, de Bruijne MC, de Keizer B, et al. The incidence, root-causes, and outcomes of adverse events in surgical units: implication for potential prevention strategies. Patient Saf Surg 2011;**5**:13.
8. Bates DW, Cullen DJ, Laird N, et al. Incidence of adverse drug events and potential adverse drug events. Implications for prevention. ADE Prevention Study Group. JAMA 1995;**274**(1):29-34.
9. Dequito AB, Mol PG, van Doormaal JE, et al. Preventable and non-preventable adverse drug events in hospitalized patients: a prospective chart review in the Netherlands. Drug Saf 2011;**34**(11):1089-100.
10. Burke JP. Infection control - a problem for patient safety. N Engl J Med 2003;**348**(7):651-6.
11. Pittet D, Donaldson L. Clean care is safer care: the first global challenge of the WHO World Alliance for Patient Safety. Am J Infect Control 2005;**33**(8):476-9.
12. World Health Organization. Chapter 3. The burden of endemic health care-associated infection in high-income countries. Report on the burden of endemic health care-associated infection worldwide. Geneva: WHO Press, 2011:12-15.
13. Donaldson L. Dirty hands: the human cost. CMO Annual Report 2006 2006.
14. World Health Organization. World Alliance for Patient Safety. The Global Patient Safety Challenge 2005–2006. Clean Care is Safer Care. Geneva, 2005.
15. Erasmus V, Daha TJ, Brug H, et al. Systematic review of studies on compliance with hand hygiene guidelines in hospital care. Infect Control Hosp Epidemiol 2010;**31**(3):283-94.
16. van de Mortel TF KS, Progano T, Sansoni J. A comparison of the hand hygiene knowledge, beliefs and practices of Italian nursing and medical students. J Adv Nurs 2012;**68**(3):569-79.
17. Erasmus V, Brouwer W, van Beeck EF, et al. A qualitative exploration of reasons for poor hand hygiene among hospital workers: lack of positive role models and of convincing evidence that hand hygiene prevents cross-infection. Infect Control Hosp Epidemiol 2009;**30**(5):415-9.
18. Lankford MG, Zembower TR, Trick WE, et al. Influence of role models and hospital design on hand hygiene of healthcare workers. Emerg Infect Dis 2003;**9**(2):217-23.
19. Paterson-Brown S. Improving patient safety through education. BMJ 2011;**342**:d214.
20. Snow M, White GL, Jr., Alder SC, et al. Mentor's hand hygiene practices influence student's hand hygiene rates. Am J Infect Control 2006;**34**(1):18-24.
21. Wakefield JG, McLaws ML, Whitby M, et al. Patient safety culture: factors that influence clinician involvement in patient safety behaviours. Qual Saf Health Care 2010;**19**(6):585-91.
22. Pittet D. Improving compliance with hand hygiene in hospitals. Infect Control Hosp Epidemiol 2000;**21**(6):381-6.
23. Whitby M, Pessoa-Silva CL, McLaws ML, et al. Behavioural considerations for hand hygiene practices: the basic building blocks. J Hosp Infect 2007;**65**(1):1-8.
24. O'Boyle CA, Henly SJ, Larson E. Understanding adherence to hand hygiene recommendations: the theory of planned behavior. Am J Infect Control 2001;**29**(6):352-60.
25. Jenner EA, Watson PW, Miller L, et al. Explaining hand hygiene practice: an extended application of the Theory of Planned Behaviour. Psychol Health Med 2002;**7**:311-26.

For peer review only - http://bmjopen.bmj.com/site/about/guidelines.xhtml

26. Sax H, Uckay I, Richet H, et al. Determinants of good adherence to hand hygiene among healthcare workers who have extensive exposure to hand hygiene campaigns. Infect Control Hosp Epidemiol 2007;**28**(11):1267-74.
27. De Wandel D, Maes L, Labeau S, et al. Behavioral determinants of hand hygiene compliance in intensive care units. Am J Crit Care 2010;**19**(3):230-9.
28. White KM, Jimmieson NL, Obst PL, et al. Using a theory of planned behaviour framework to explore hand hygiene beliefs at the '5 critical moments' among Australian hospital-based nurses. BMC Health Serv Res 2015;**15**:59.
29. Ajzen J. The theory of planned behavior. Organizational behavior and human decision processes 1991;**50**:179-211.
30. Hunt DC, Mohammudally A, Stone SP, et al. Hand-hygiene behaviour, attitudes and beliefs in first year clinical medical students. J Hosp Infect 2005;**59**(4):371-3.
31. Polacco MA, Shinkunas L, Perencevich EN, et al. See one, do one, teach one: hand hygiene attitudes among medical students, interns, and faculty. Am J Infect Control 2015;**43**(2):159-61.
32. van de Mortel TF, Apostolopoulou E, Petrikkos G. A comparison of the hand hygiene knowledge, beliefs, and practices of Greek nursing and medical students. Am J Infect Control 2010;**38**(1):75-7.
33. van de Mortel TF. Development of a questionnaire to assess health care students' hand hygiene knowledge, beliefs and practices. Aust J Adv Nurs 2009;**26**(3):9-16.
34. Humphreys H, Richards J. Undergraduate and postgraduate medical education on the prevention and control of healthcare-associated infection. Int J Infect Control 2011; **v7**::i2.
35. O'Boyle C A, Henly SJ, Duckett LJ. Nurses' motivation to wash their hands: a standardized measurement approach. Appl Nurs Res 2001;**14**(3):136-45.
36. Bronfenbrenner U. *The ecology of human development*. Cambridge, MA: Harvard University Press, 1979.
37. Verplanken B, Orbell S. Reflections on Past Behavior: A Self-Report Index of Habit Strength. Journal of Applied Social Psychology;**33**(6):1313-30.
38. van der Velden LF, Hingstman L, Heiligers PJ, et al. [Increasing number of women in medicine: past, present and future] Toenemend percentage vrouwen in de geneeskunde: verleden, heden en toekomst. Ned Tijdschr Geneeskd 2008;**152**(40):2165-71.
39. van de Mortel, T. Faking it: social desirability response bias in self-report research. Austr J Adv Nurs 2008; **25**(4):40-48.
40. Nieva VF, Sorra J. Safety culture assessment: a tool for improving patient safety in healthcare organizations. Qual Saf Health Care 2003;**12 Suppl 2**:ii17-23.
41. Fuller C, Besser S, Savage J, et al. Application of a theoretical framework for behavior change to hospital workers' real-time explanations for noncompliance with hand hygiene guidelines. Am J Infect Control 2014;**42**(2):106-10.
42. von Lengerke T, Lutze B, Graf K, et al. Psychosocial determinants of self-reported hand hygiene behaviour: a survey comparing physicians and nurses in intensive care units. J Hosp Infect 2015;**91**(1):59-67.
43. Feather A, Stone SP, Wessier A, et al. 'Now please wash your hands': the handwashing behaviour of final MBBS candidates. Journal of Hospital Infection 2000;**45**(1):62-64.
44. Wong TW, Tam WW. Handwashing practice and the use of personal protective equipment among medical students after the SARS epidemic in Hong Kong. Am J Infect Control 2005;**33**(10):580-6.
45. Pittet D, Simon A, Hugonnet S, et al. Hand hygiene among physicians: performance, beliefs, and perceptions. Ann Intern Med 2004;**141**(1):1-8.
46. Verplanken B, Aarts H. Habit, Attitude and Planned Behaviour: Is Habit an Empty Construct or an Interesting Case of Goal-Directed Automaticity? Eur Rev Soc Psychol 1999;**10**:101-34.
47. Rome M, Sabel A, Price CS, et al. Hand hygiene compliance. J Hosp Infect 2007;65(2):173.

For peer review only

For peer review only - http://bmjopen.bmj.com/site/about/guidelines.xhtml

48. Walton MM. Hierarchies: the Berlin Wall of patient safety. Qual Saf Health Care 2006;**15**(4):229-30.
49. Mann CM, Wood A. How much do medical students know about infection control? J Hosp Infect 2006;**64**(4):366-70.
50. Varkey P, Karlapudi S, Rose S, et al. A patient safety curriculum for graduate medical education: results from a needs assessment of educators and patient safety experts. Am J Med Qual 2009;**24**(3):214-21.
51. Gollwitzer PM, Sheeran P. Implementation intentions and goal achievement: a meta-analysis of effects and processes. Adv Exp Soc Psychol 2006;**38**:69-119.
52. Sniehotta FF, Scholz U, Schwarzer R. Bridging the intention-behaviour gap: Planning, self-efficacy, and action control in the adoption and maintenance of physical exercise. Psychol Health 2005;**20**(2):143-60.
53. Erasmus V, Kuperus MN, Richardus JH, et al. Improving hand hygiene behaviour of nurses using action planning: a pilot study in the intensive care unit and surgical ward. J Hosp Infect 2010;**76**(2):161-4.

For peer review only

Table 1: Constructs of the questionnaire on behavioural determinants of hand hygiene with example questions and internal consistency (Cronbach's α)

Construct	# items	Mean (SD)	Cronbach's α	Example
**Knowledge**	5	4.3 (.78)	-	5 true/false questions about factual knowledge
**Risk perception**:				
Chance	3	5.6 (1,7)	0.76	How big is the chance that an infection will occur
Severity self	1	5.6 (2.3)	-	How severe will the consequences of an infection be for myself
Severity patient	1	7.2 (1.5)	-	How severe will the consequences of an infection be for my patient
**Attitudes**:				
Beliefs about hand hygiene	8	5.3 (.81)	0.76	Hand hygiene is something I find important
Perceived outcomes	5	5.0 (.98)	0.78	If I follow that hand hygiene guidelines my patients will develop fewer infections
**Social norms**:				
Referent beliefs	4	4.7 (1.2)	0.91	My superior thinks that I should always follow the hand hygiene guidelines
Descriptive norm	8	3.5 (.81)	0.73	My colleagues always follow the hand hygiene guidelines
**Self-efficacy**	11	4.8 (.94)	0.89	I am certain that I will be able to follow the hand hygiene guidelines
**Habit**	12	4.7 (1.1)	0.95	Following the hand hygiene guidelines is something I do without thinking about it

For peer review only

Table 2: Behavioural correlates of hand hygiene compliance of medical students (n=313)

	Model 1		Model 2			Model 3		
	β	R^2	B	R^2	$R^{2change}$	β	R^2	$R^{2change}$
		.043		.270	.189		.401	.131
Knowledge	.081		.044			.063		
Risk perception:								
Chance	.101		.049			.010		
Severity self	.105		.041			.002		
Severity patient	.102		.036			.019		
Attitude:								
Beliefs			.103			-.026		
Perceived outcomes			.231***			.174**		
Social norms:								
Referent beliefs			.003			-.001		
Descriptive norm			.063			.037		
Self efficacy			.306***			.138*		
Habit						.471***		

* P<.05; ** P<.01; *** P < .001

Model 1: knowledge + risk perception

Model 2: knowledge + risk perception + attitudes + social norms + self efficacy

Model 3: knowledge + risk perception + attitudes + social norms + self efficacy + habit

For peer review only

For peer review only - http://bmjopen.bmj.com/site/about/guidelines.xhtml

Figure 1: Extended Theory of Planned Behaviour model. Constructs in gray boxes have been added.

For peer review only

For peer review only - http://bmjopen.bmj.com/site/about/guidelines.xhtml

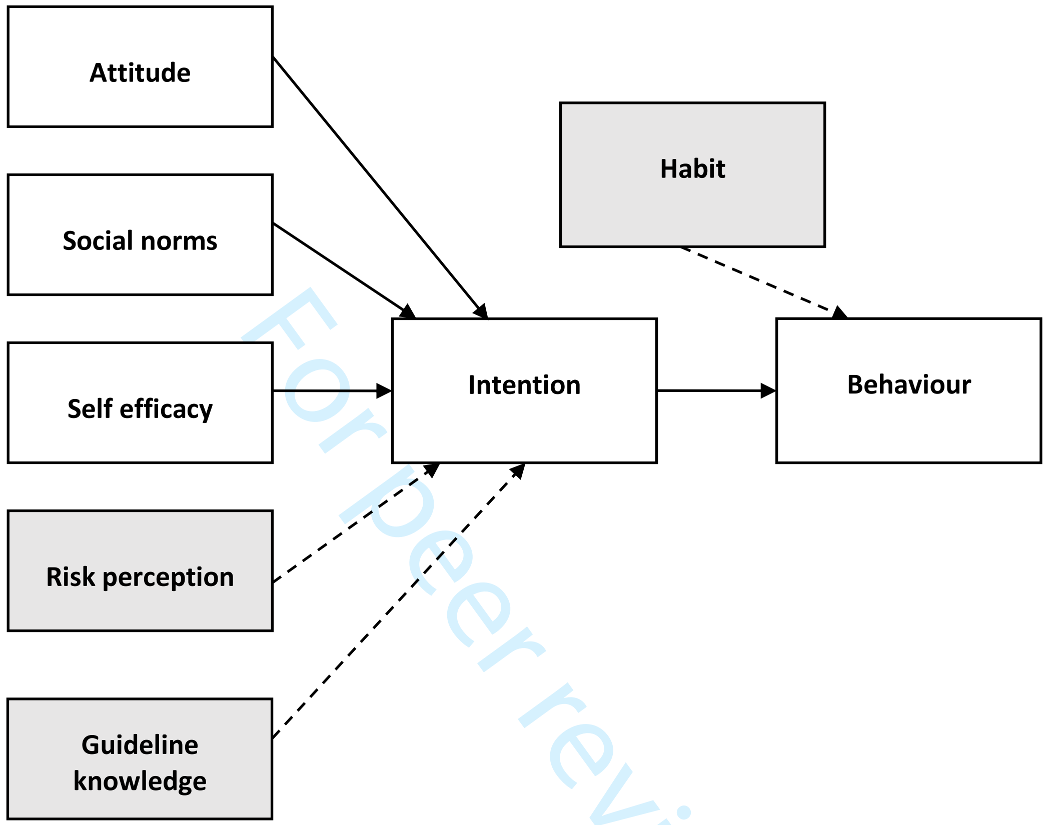

For peer review only

Reporting checklist for cross sectional study.

Based on the STROBE cross sectional guidelines.

Instructions to authors

Complete this checklist by entering the page numbers from your manuscript where readers will find each of the items listed below.

Your article may not currently address all the items on the checklist. Please modify your text to include the missing information. If you are certain that an item does not apply, please write "n/a" and provide a short explanation.

Upload your completed checklist as an extra file when you submit to a journal.

In your methods section, say that you used the STROBE cross sectional reporting guidelines, and cite them as:

von Elm E, Altman DG, Egger M, Pocock SJ, Gotzsche PC, Vandenbroucke JP. The Strengthening the Reporting of Observational Studies in Epidemiology (STROBE) Statement: guidelines for reporting observational studies.

		Reporting Item	Page Number
Title	#1a	Indicate the study’s design with a commonly used term in the title or the abstract	1
Abstract	#1b	Provide in the abstract an informative and balanced summary of what was done and what was found	2
Background / rationale	#2	Explain the scientific background and rationale for the investigation being reported	4
Objectives	#3	State specific objectives, including any prespecified hypotheses	5
Study design	#4	Present key elements of study design early in the paper	5
Setting	#5	Describe the setting, locations, and relevant dates, including periods of recruitment, exposure, follow-up, and data collection	5
Eligibility criteria	#6a	Give the eligibility criteria, and the sources and methods of selection of participants.	5

For peer review only

	#7	Clearly define all outcomes, exposures, predictors, potential confounders, and effect modifiers. Give diagnostic criteria, if applicable	6
Data sources / measurement	#8	For each variable of interest give sources of data and details of methods of assessment (measurement). Describe comparability of assessment methods if there is more than one group. Give information separately for for exposed and unexposed groups if applicable.	5-6
Bias	#9	Describe any efforts to address potential sources of bias	n/a
Study size	#10	Explain how the study size was arrived at	5
Quantitative variables	#11	Explain how quantitative variables were handled in the analyses. If applicable, describe which groupings were chosen, and why	7
Statistical methods	#12a	Describe all statistical methods, including those used to control for confounding	7
	#12b	Describe any methods used to examine subgroups and interactions	n/a
	#12c	Explain how missing data were addressed	n/a
	#12d	If applicable, describe analytical methods taking account of sampling strategy	n/a
	#12e	Describe any sensitivity analyses	n/a
Participants	#13a	Report numbers of individuals at each stage of study—eg numbers potentially eligible, examined for eligibility, confirmed eligible, included in the study, completing follow-up, and analysed. Give information separately for for exposed and unexposed groups if applicable.	8
	#13b	Give reasons for non-participation at each stage	n/a
	#13c	Consider use of a flow diagram	n/a
Descriptive data	#14a	Give characteristics of study participants (eg demographic, clinical, social) and information on exposures and potential confounders. Give information separately for exposed and unexposed groups if applicable.	8

For peer review only

	#14b	Indicate number of participants with missing data for each variable of interest	n/a
Outcome data	#15	Report numbers of outcome events or summary measures. Give information separately for exposed and unexposed groups if applicable.	8-9, 19
Main results	#16a	Give unadjusted estimates and, if applicable, confounder-adjusted estimates and their precision (eg, 95% confidence interval). Make clear which confounders were adjusted for and why they were included	n/a
	#16b	Report category boundaries when continuous variables were categorized	n/a
	#16c	If relevant, consider translating estimates of relative risk into absolute risk for a meaningful time period	n/a
Other analyses	#17	Report other analyses done—e.g., analyses of subgroups and interactions, and sensitivity analyses	n/a
Key results	#18	Summarise key results with reference to study objectives	8
Limitations	#19	Discuss limitations of the study, taking into account sources of potential bias or imprecision. Discuss both direction and magnitude of any potential bias.	9-10
Interpretation	#20	Give a cautious overall interpretation considering objectives, limitations, multiplicity of analyses, results from similar studies, and other relevant evidence.	13
Generalisability	#21	Discuss the generalisability (external validity) of the study results	11-12
Funding	#22	Give the source of funding and the role of the funders for the present study and, if applicable, for the original study on which the present article is based	14

The STROBE checklist is distributed under the terms of the Creative Commons Attribution License CC-BY. This checklist was completed on 12. December 2018 using http://www.goodreports.org/, a tool made by the EQUATOR Network in collaboration with Penelope.ai

For peer review only
